# Synthesis of natural product hybrids by the Ugi reaction in complex media containing plant extracts

**DOI:** 10.1038/s41598-022-19579-6

**Published:** 2022-09-16

**Authors:** Keisuke Tomohara, Nao Ohashi, Tatsuya Uchida, Takeru Nose

**Affiliations:** 1 Faculty of Arts and Science, Kyushu University, 744 Motooka, Nishi-ku, Fukuoka, 819-0395 Japan; 2 Graduate School of Science, Kyushu University, 744 Motooka, Nishi-ku, Fukuoka, 819-0395 Japan; 3 International Institute for Carbon-Neutral Energy Research, Kyushu University, 744 Motooka, Nishi-ku, Fukuoka, 819-0395 Japan

**Keywords:** Plant sciences, Chemical libraries, Diversity-oriented synthesis, Isolation, separation and purification, Molecular engineering in plants

## Abstract

Plant extracts are rich in a wide variety of molecules with diverse biological activities. Chemical engineering of plant extracts has provided a straightforward and simultaneous synthetic route for artificial molecules derived from plant products. This study achieved the synthesis of 13 natural product-like molecules by the Ugi multicomponent reaction using plant extracts as substrates. In particular, the engineering of a mixture of plant extracts demonstrated a unique synthetic route to a series of natural product hybrids, whereby otherwise unencountered naturally occurring molecules of different origins were chemically hybridized in complex media. Even though these reactions took place in complex media containing plant extracts, the well-designed process achieved a good conversion efficiency (~ 60%), chemoselectivity, and reproducibility. Additionally, some of the Ugi adducts exhibited promising inhibitory activity toward protease.

## Introduction

Plants produce hundreds or even thousands of structurally diverse and complex chemicals to meet their nutritional, metabolic, defensive, and reproductive requirements^1–3^. The chemical composition of plants differs depending on species, life stages, habitats, and environments and reflects survival strategies that have developed over millions of years of survival competition, selection, and evolution^2,3^. Thus, plants have been and will continue to be useful suppliers of various chemicals beyond our expectations and imagination. The unique chemical production process of plants has inspired chemists to explore and utilize plant products as reliable starting points for the discovery and development of pharmaceuticals and agrochemicals^4,5^. Indeed, scores of approved pharmaceuticals and agrochemicals, and the candidates thereof, originate from plant products^6,7^. However, after more than a century of exhaustive studies, it is now increasingly difficult to identify unexplored chemicals in plants^8^. In turn, chemists have explored the biosynthetic mechanism of plants and manipulated the corresponding pathways and enzymes to acquire unusual or unexplored biosynthetic molecules^9,10^. Chemists have also designed bioinspired artificial molecules and synthesized them in flasks from readily available small petrochemicals or “isolated” natural products, although significant effort is needed to create diverse and elaborate chemicals similar to natural products.

**Figure 1:**
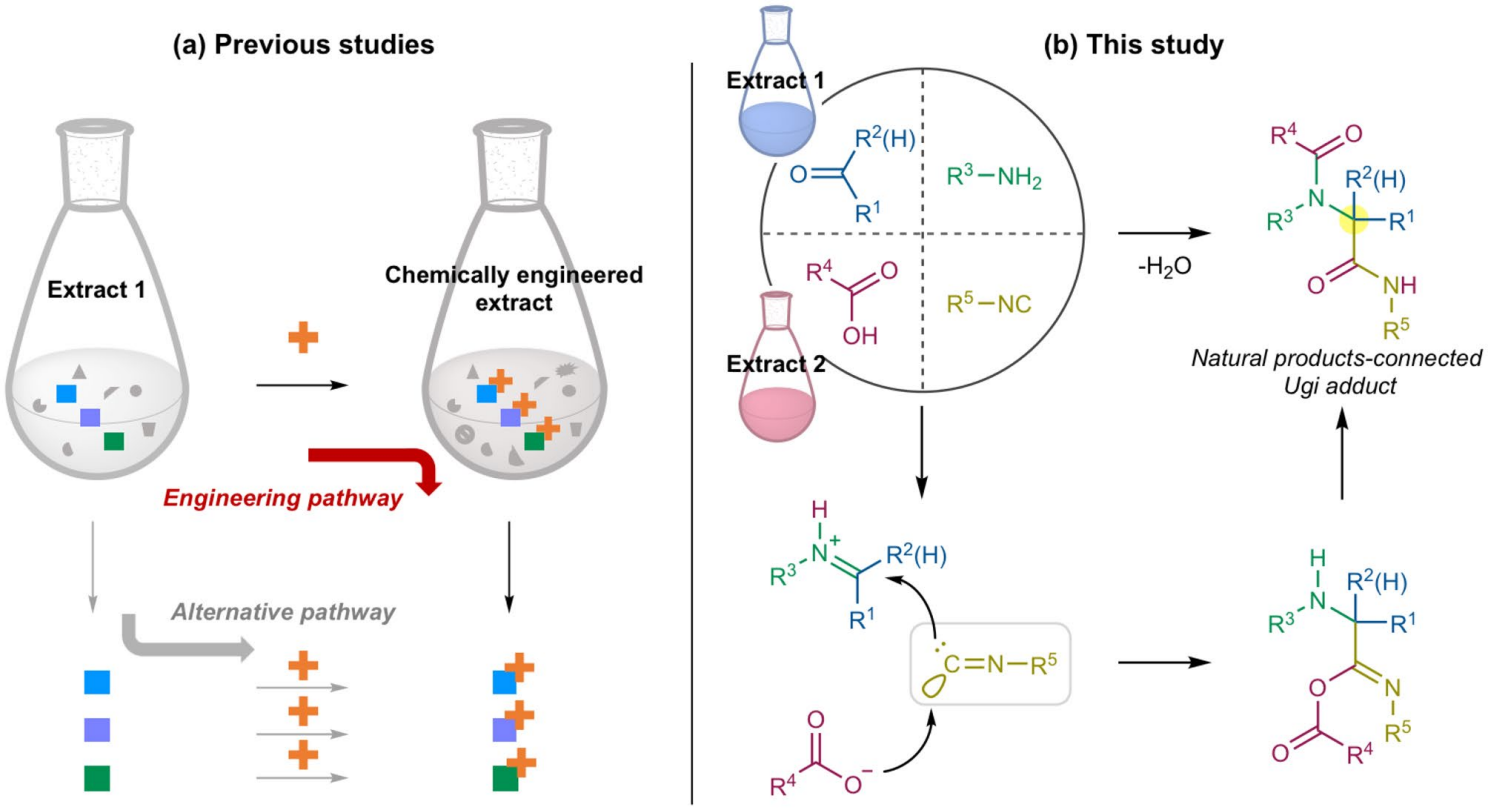
(**a**) Chemical engineering of natural product extracts reported in the literature. (**b**) Chemical hybridization of natural products by the Ugi-4CR in this study.

Another approach for obtaining artificial molecules derived from natural products has been explored by chemical engineering of natural product extracts (Fig. 1a, shown in red)^5,11^. This method enables natural product-like molecules to be designed and synthesized directly from naturally occurring chemicals in readily available natural product extracts. An advantage of this method is that if the extracts possess several reactive molecules, this method simultaneously provides a set of molecules derived from natural products in a single synthetic operation^12–14^. However, treating natural product extracts with synthetic reagents often causes unfavorable events such as unintended reactions, decomposition of the extract components, and remaining reagents, and thereby the resulting engineered mixtures tend to become much more complicated^15^. Consequently, subsequent isolation of products from the reaction mixtures requires tedious separation, purification, structural determination, and dereplication. Moreover, the guidance of biological activity is sometimes required^16^, as is observed in traditional natural product discoveries. A limitation of this synthetic method is also found in the fact that all previous engineering has simply modified single functional groups or moieties of natural products in the extracts. These parallel modifications of natural products can also be replaced by a stepwise operation involving isolation of a set of targeted molecules from extracts and subsequent chemical manipulation of individual molecules (Fig. 1a, shown in grey). In several cases, such a traditional sequential approach may be easier to operate than the chemical engineering approach. Thus, this newborn method remained far from broad attention and application.

Here, this study achieved the design and synthesis of 13 natural plant product-like molecules by the Ugi four-component reaction (Ugi-4CR) using natural plant extracts as substrates, without causing unnecessary complications to the reaction mixture. In particular, the engineering using a mixture of the methanol extract of *Curcuma zedoaria* and castor oil fatty acids successfully provided a synthetic route to a series of natural product hybrids, whereby otherwise unencountered naturally occurring molecules of different species were chemically hybridized in a complex mixture of natural plant extracts.

## Results and discussion

### Designing a chemical engineering scheme based on the racemic Ugi reaction

Chemical hybridization of plant products was realized by chemical engineering of plant extracts using the racemic Ugi four-component reaction (Ugi-4CR), wherein four different types of functional group components, namely, an aldehyde or ketone, a primary amine, a carboxylic acid, and an isocyanide, assemble into an α-acylamino amide (Fig. 1b)^17,18^. An isocyanide possessing both nucleophilic and electrophilic properties serves as a coupling reagent for the plant products. Typically, a one-pot procedure with a stoichiometric mixture of substrate components yields an Ugi adduct quantitatively, with water as the sole by-product^19^. The high step- and atom-economy of the Ugi-4CR would be suitable for executing the chemical engineering of natural product extracts without causing unnecessary complication to the reaction mixture^20–23^. Additionally, the natural abundance of aldehydes, ketones, and carboxylic acids in plants^4,24,25^ motivated us to employ plant extracts as substrates of the Ugi-4CR.

First, using model compounds, the substrate scope of the Ugi-4CR was briefly investigated (Supplementary Fig. S1). The reaction time was fixed for 7 days for all entries. Importantly, the reaction temperature was set at room temperature and four substrates were mixed stoichiometrically, in order to avoid undesired side reactions and/or decompositions of constituents in extracts. In summary, this mild reaction conditions exhibited broad scope and afforded Ugi adducts in moderate to high yields (11 examples, 45–93% yields), and the Ugi-4CR was thus found to be suitable for chemical engineering of plant extracts. While some of the substrates required only a couple of days to complete the reaction, other substrates exhibited slow conversions (Supplementary Fig. S1, entries **1a**, **1b** in TFE, **1f**, and **1h**). Thus, the reaction time for the following Ugi reaction using plant extracts was set to 7 days.

### Synthesis of an Ugi adduct derived from an extract of *Zanthoxylum piperitum*

**Figure 2:**
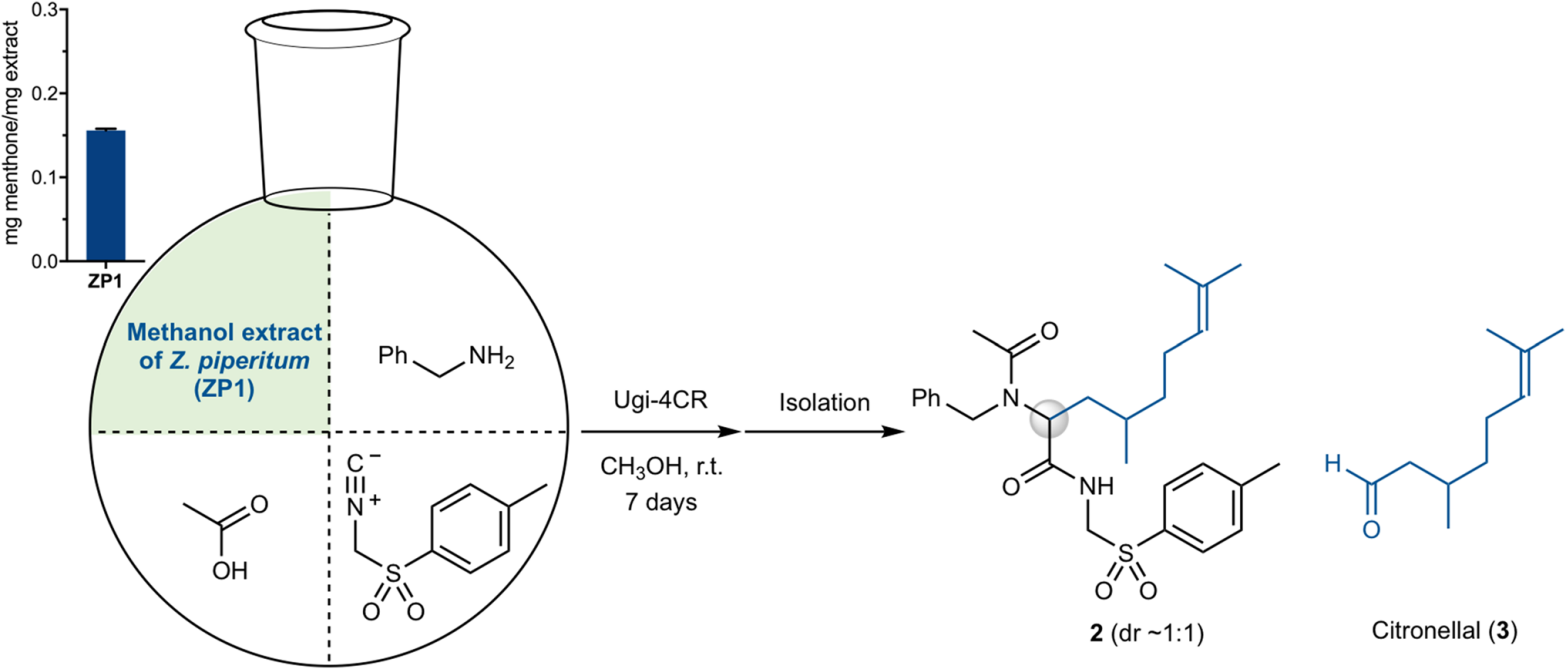
Synthesis of **2** by the Ugi-4CR of the methanol extract of *Z. piperitum* (ZP1) in the presence of benzylamine, acetic acid, and TosMIC. The inset shows the carbonyl content of the ZP1 extract.

Under the optimized reaction conditions, the first chemical engineering of a plant extract was performed using the methanol extract of the commercially available pericarp of *Zanthoxylum piperitum* (ZP1) as the carbonyl component of the Ugi-4CR (Fig. 2). The content of the carbonyl compounds in the ZP1 extract was roughly estimated to be 0.156 ± 0.002 mg menthone per mg extract by colorimetric quantification using 2,4-dinitrophenylhydrazonate.^26^ The ZP1 extract (1.32 g) was mixed with excess benzylamine, acetic acid, and *p*-toluenesulfonylmethyl isocyanide (TosMIC) in methanol. After stirring for 7 days, the crude mixture was repeatedly separated and purified by silica gel column chromatography and preparative thin layer chromatography (TLC) to isolate the novel Ugi adduct **2** (5.28 mg), which was found to derive from citronellal (**3**) in ZP1^27^. The structure of **2** was well-characterized by a combination of ^1^H NMR, ^1^H–^1^H COSY, ^13^C NMR, DEPT, HMQC, HMBC, IR, and MS analyses. During the separation and purification stages, the characteristic ^1^H NMR signals derived from the tosyl and benzyl groups in **2** and the UV absorbance were effectively used to identify the product. The ease of handling of this process is in sharp contrast to the tediousness of isolating **3** from the ZP1 extract and the subsequent chemical manipulation processes because of the low content of **3** in the ZP1 extract and the absence of UV absorption at 254 nm.

### Simultaneous synthesis of Ugi adducts derived from extracts of the genus *Curcuma*

**Figure 3:**
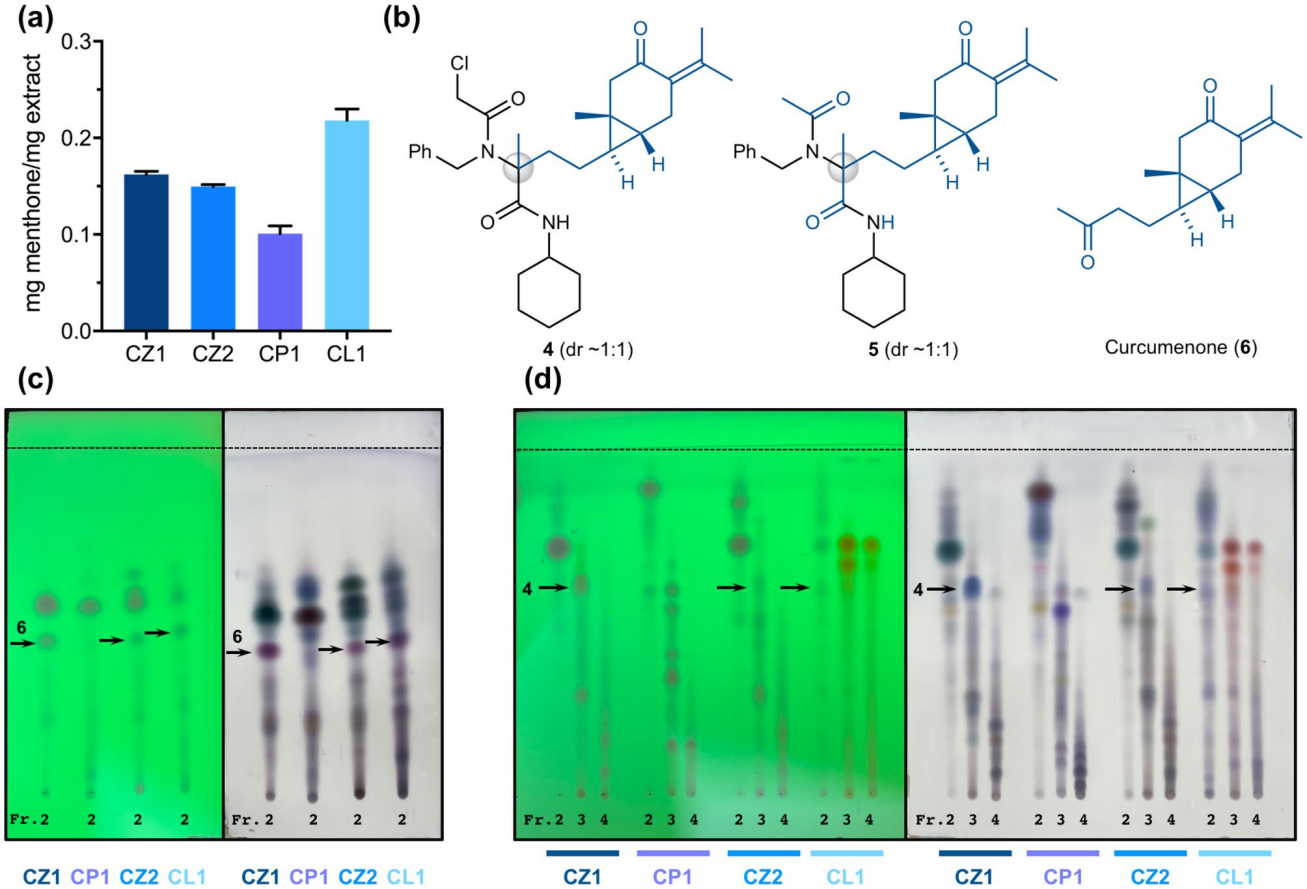
(**a**) Carbonyl content of the extracts. (**b**) Structures of Ugi adducts **4** and **5**, and curcumenone (**6**). (**c**) TLC of fraction No. 2 of the untreated extracts. The TLC was developed with hexane/ethyl acetate (4:1) and then visualized by UV light at 254 nm (i) or by staining with an acidic solution of *p*-anisaldehyde (ii). (**d**) TLC of fractions No. 2–4 of the engineered mixtures. The TLC was developed with 1,2-dichloroethane/ethyl acetate (19:6) and then visualized by UV light at 254 nm (i) or by staining with an acidic solution of *p*-anisaldehyde (ii). The dashed lines in (**c**) and (**d**) indicate the solvent front.

Then, chemical engineering of plant extracts was performed using the commercially available dried rhizome powder of *Curcuma zedoaria* from China (CZ1) as the carbonyl component of the Ugi-4CR. The carbonyl content of the methanol extract of CZ1 was estimated to be 0.162 ± 0.003 mg menthone per mg extract (Fig. 3a), and a stoichiometric mixture of the CZ1 extract, benzylamine, chloroacetic acid, and cyclohexyl isocyanide was subjected to the Ugi-4CR conditions. Based on the results presented in Supplementary Fig. S1, the most reactive acid, chloroacetic acid, was used as the acid component. The resulting engineered mixture and the untreated CZ1 extract were then roughly divided into six fractions by silica gel column chromatography, and their chemical composition was analyzed by TLC (Supplementary Fig. S2). The result showed that most spots (constituents) in the CZ1 extract remained unchanged under the engineering conditions, and clearly highlighted a disappeared spot (substrate) as well as newly appeared spots (products to be isolated). Repeated separation and purification of the engineered mixture resulted in the successful identification of two novel Ugi adducts **4** and **5**, both of which were found to derive from curcumenone (**6**) in CZ1 (Fig. 3b)^28^. The acetyl component in **5** was assumed to derive from CZ1. Both products had a newly constructed tetra-substituted stereocenter and existed as a 1:1 mixture of diastereomers. The conversion efficiency in this engineering procedure was roughly estimated to be 58% based on the content of **6** in the starting CZ1 extract, which was determined through tedious separation and purification processes (Table 1, entry 1). This conversion efficiency was quite satisfactory considering that the present reaction was performed in the presence of many unidentified chemicals, indicating the high chemoselectivity of the Ugi-4CR-based engineering process. The same process was also applied to the methanol extract of the dried rhizome powder of *C. zedoaria* from Japan (CZ2) to provide **4** in a slightly reduced yield (28%, entry 2, Supplementary Fig. S3). By contrast, no Ugi adduct was obtained from the reaction with *C. phaeocaulis* (CP1) because this extract did not contain curcumenone or other reactive aldehydes or ketones (entry 3). As expected, the chemical composition of the engineered CP1 mixture apparently remained unchanged under the present Ugi-4CR conditions (Supplementary Fig. S4). Reaction of the methanol extract of *C. longa* (CL1) containing curcumenone also afforded the Ugi adduct **4** with a conversion efficiency of 49% (entry 4, Supplementary Fig. S5), in good agreement with the engineering results for CZ1 (entry 1). The similar conversion efficiencies, regardless of plant origin, demonstrated the reproducibility of the present engineering method (Supplementary Fig. S6). The extracts containing curcumenone, CZ1, CZ2, and CL1, had different chemical composition, as shown by the corresponding TLC analyses (Fig. 3c,d); however, all engineering processes similarly provided the Ugi adducts, demonstrating the robustness of the proposed method. Of note, the isolated curucumenone (**6**) was found to be unstable and gradually decomposed. Thus, the present engineering method enabled the direct chemical modification of plant products that would otherwise be inaccessible.

**Table 1:** Ugi-4CR using the extracts of the genus *Curcuma*.

Entry	Plant origin (country)	Yield of Ugi adduct (mg)^a^	Conversion efficiency (%)^b^
1 (CZ1)	*C. zedoaria* (Japan)	**4** (37.5 mg), **5** (1.99 mg)	58
2 (CZ2)	*C. zedoaria* (China)	**4** (4.03 mg)	28
3 (CP1)	*C. paeocaulis* (China)	–^c^	–
4 (CL1)	*C. longa* (Japan)	**4** (16.2 mg)	49

^a^Isolated yield. ^b^Estimated from the curcumenone content of the extract. ^c^Not obtained.

### Fluorescence-guided isolation strategy of an Ugi adduct derived from a plant extract

**Figure 4:**
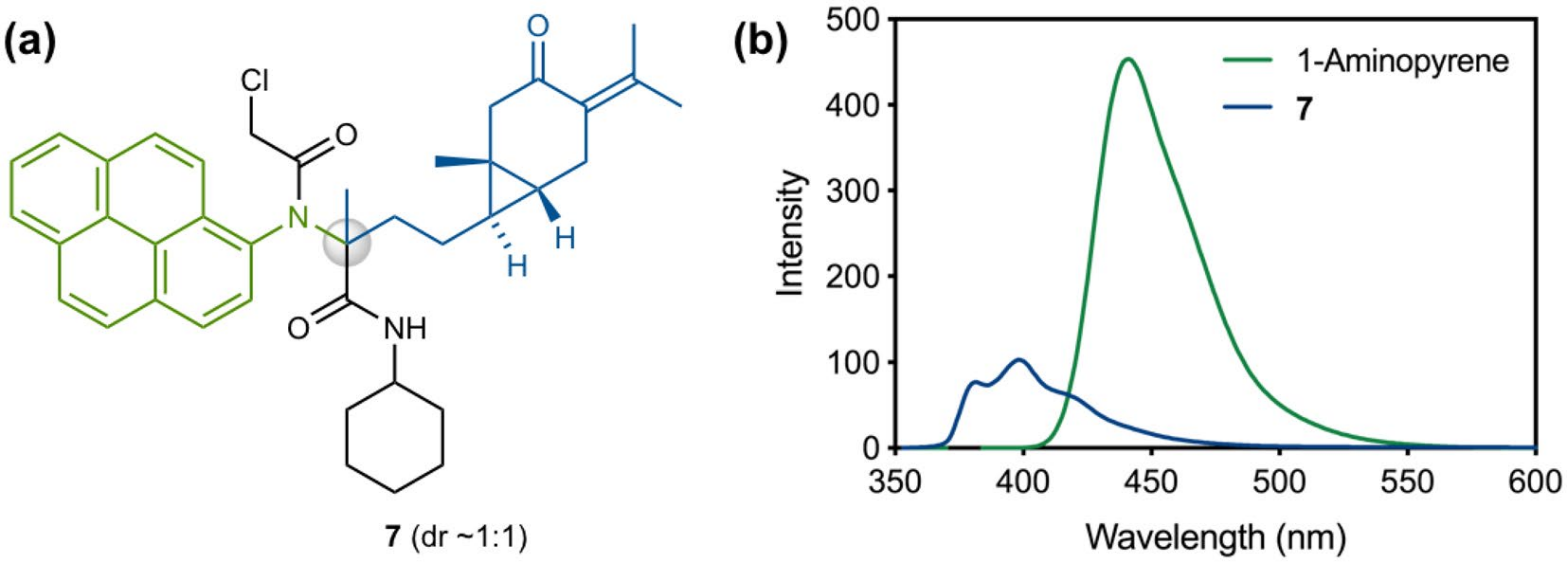
(**a**) Structure of **7**. (**b**) Fluorescence spectra of 1-aminopyrene and **7** (1 μM) in DMSO with 342 nm excitation.

Instead of using benzylamine, a similar engineering of the CZ1 extract was performed in the presence of the fluorescent 1-aminopyrene to provide the expected Ugi adduct **7**, where the benzyl group of **6** was replaced with the 1-pyrenyl motif (Fig. 4a). Upon excitation at 342 nm, product **7** exhibited fluorescence, where the maximum emission wavelength (${\uplambda }_{max}^{em}$) in DMSO was significantly blue-shifted from that of 1-aminopyrene (${\uplambda }_{max}^{em}$ = 441 nm) (Fig. 4b)^29,30^. This blue-shift of ${\uplambda }_{max}^{em}$ associated with 1-pyrenyl amide formation enabled facile identification of the Ugi adduct **7** from the crude reaction mixture by irradiation with a handy UV lamp (365 nm), although the fluorescence intensity of **7** was lower than that of 1-aminopyrene. This engineering example showed that the fluorescence-guided isolation strategy^31^ can facilitate identification of plant products that may have otherwise been missed, and that the simple replacement of amine and isocyanide would provide rapid access to a series of analogues.

### Chemical composition of castor oil fatty acids

Another engineering was achieved using castor oil fatty acids (CO-FA), prepared from the seed oil of *Ricinus communis*, as the carboxylic acid component of the Ugi-4CR. Compared to other plant oils, castor oil possesses a high content of ricinoleic acid and is thus highly soluble in alcohols. These unique properties of CO-FA prompted us to use it in the Ugi-4CR-based engineering process. The acid content of CO-FA was deduced from the given neutralization number (183.6 mg KOH g^−1^), and a stoichiometric mixture (0.5 mmol) of CO-FA, (±)-citronellal, benzylamine, and cyclohexyl isocyanide was subjected to the Ugi-4CR conditions. The engineering proceeded smoothly and the subsequent two steps of separation and purification of the engineered mixture by silica gel column chromatography and preparative TLC revealed four different types of Ugi adducts **8a**–**8d**, which were found to be derived from (*R*)-ricinoleic acid, dimeric (*R*)-ricinoleic acid^32^, oleic acid, and linoleic acid in CO-FA, respectively (Table 2)^33^. The mixture also gave Ugi adduct **9**, which was likely to be derived from (*R*)-ricinoleic acid and benzaldehyde in CO-FA. Taken together, CO-FA engineering using the Ugi-4CR afforded five novel Ugi adducts in a single synthetic operation. All the Ugi adducts were easily isolated based on their UV-absorbing properties and structurally well-characterized by a set of NMR analyses. The production ratio (wt%) of Ugi adducts **8a**, **9**, **8c**, and **8d** approximately corresponded to the reported content of (*R*)-ricinoleic acid, oleic acid, and linoleic acid in CO-FA, respectively (Table 2)^33^. These results indicated that the present engineering method could be used to determine the composition of acids in CO-FA samples.

**Table 2:** Ugi adducts **8a**–**8d** and **9** derived from CO-FA.


Fatty acids in CO-FA			R	Ugi adduct	Yield		Conversion efficiency (%)
					mg	Ratio (wt%)	
(*R*)-Ricinoleic acid	18:1^Δ9^	90%^a^	(*R*,*Z*)-12-Hydroxy-9-octadecenoyl	**8a**	143.44^b^	84	44
			-	**9**	4.20^b^		1.4
Dimeric (*R*)-ricinoleic acid			(*R*,*Z*)-12-(((*R*,*Z*)-12-Hydroxy-9-octadecenoyl)oxy)-9-octadecenoyl	**8b**	5.29^b^	3	1.1
Oleic acid	18:1^Δ9^	3%^a^	(*Z*)-9-Octadecenoyl	**8c**	11.04^c^	6	3.5
Linoleic acid	18:2^Δ9,12^	4%^a^	(*Z*,*Z*)-9,12-Octadecadienoyl	**8d**	11.00^c^	6	3.5
Total					174.97	100	54

^a^Data (wt%) from Ref.^33^. ^b^Isolated yield. ^c^Estimated yield from ^1^H NMR analysis of a mixture of **8c** and **8d**.

### Synthesis of plant product hybrids

**Figure 5:**
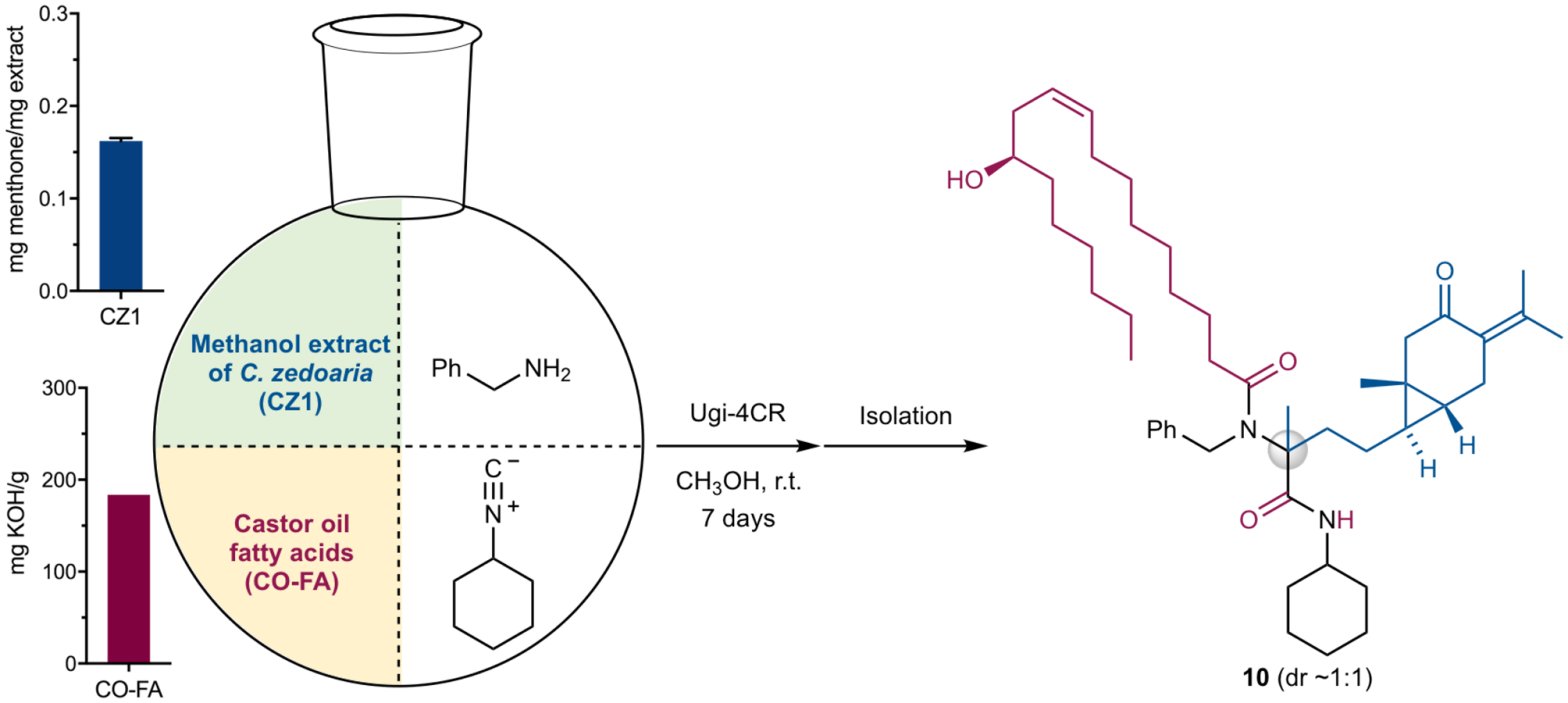
Synthetic of **10** by the Ugi-4CR using a mixture of CZ1 and CO-FA in the presence of benzylamine and cyclohexyl isocyanide. The inserts show the carbonyl content of the CZ1 extract and the neutralization number of CO-FA.

**Figure 6:**
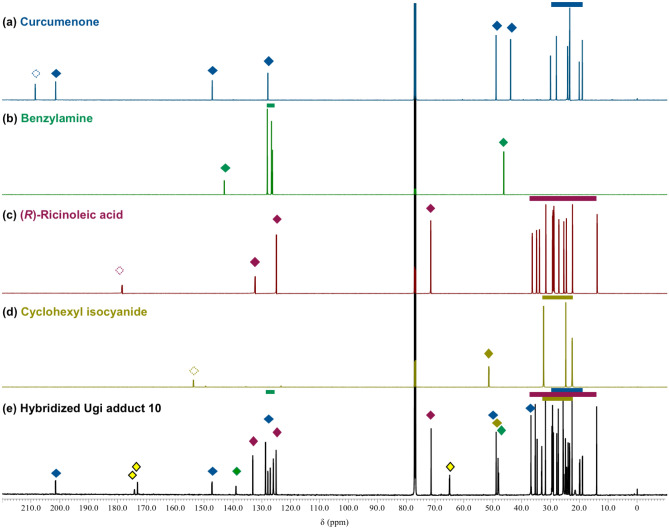
^13^C NMR spectra (151 MHz) of (**a**) curcumenone, (**b**) benzylamine, (**c**) ricinoleic acid, (**d**) cyclohexyl isocyanide, and (**e**) hybridized Ugi adduct **10** in CDCl_3_. Units of chemical shift (δ) are ppm relative to residual chloroform (77.16 ppm) as an internal standard.

**Figure 7:**
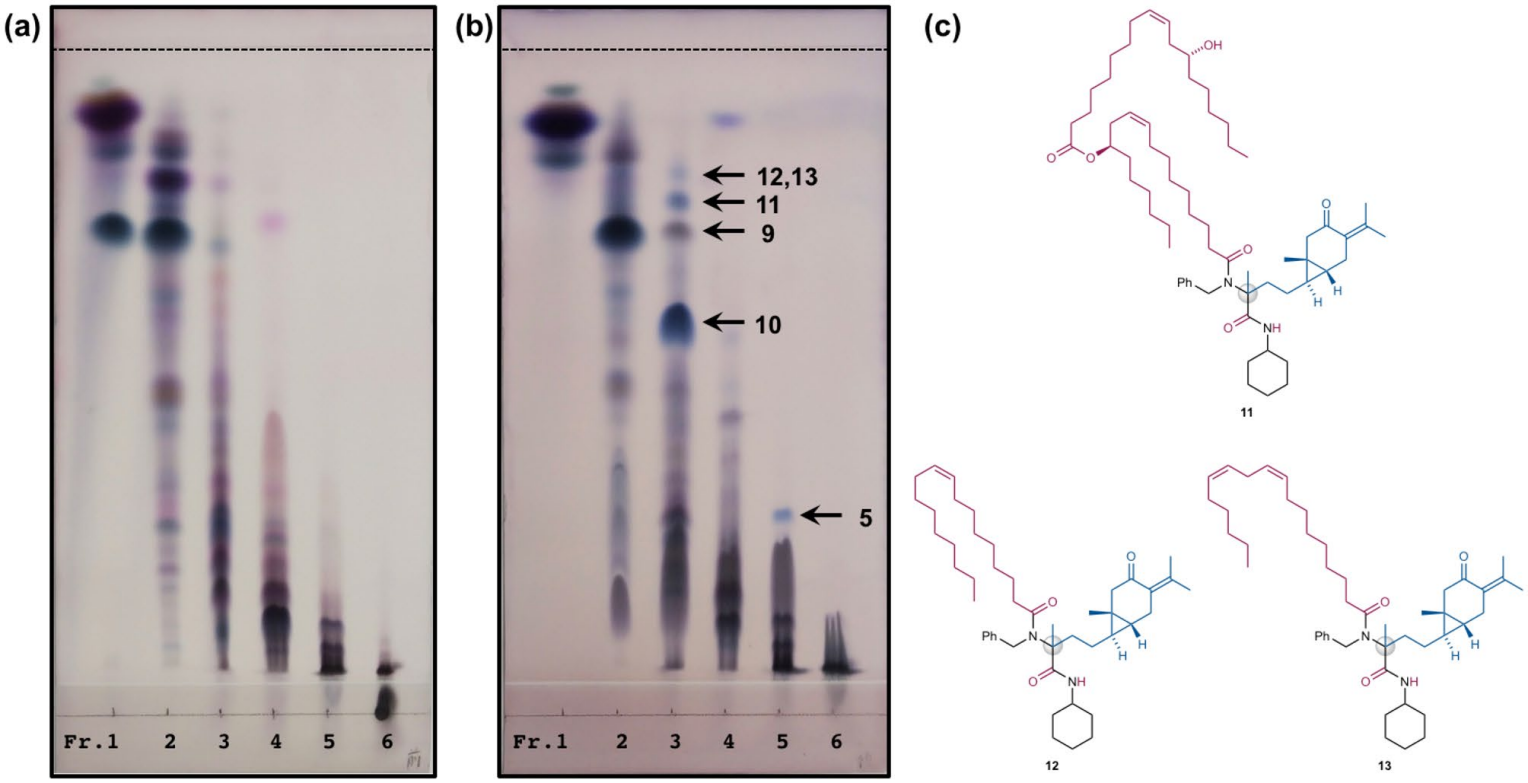
(**a**) TLC of the fractionated starting extract of *C. zedoaria* (CZ1). (**b**) TLC of the fractionated engineered mixture. Both TLCs were developed with CHCl_3_/ethyl acetate (19:6) and then visualized by staining with an acidic solution of *p*-anisaldehyde. The upper dashed line indicates the solvent front. (**c**) Structures of the hybridized Ugi adducts **11**–**13**.

Our goal of synthesizing plant product hybrids was finally achieved by the Ugi-4CR using a mixture of the methanol extract of *C. zedoaria* (CZ1) and castor oil fatty acids (CO-FA) as substrates in the presence of benzylamine and cyclohexyl isocyanide (Fig. 5). A stoichiometric mixture of the four substrate components in methanol was stirred at room temperature for 7 days to give the engineered mixture. Separation and purification of the engineered mixture identified the novel Ugi adduct **10** (53.8 mg, 57%) as a 1:1 mixture of diastereomers, which was assigned to be a hybrid-type molecule of curcumenone (**6**) in CZ1 and ricinoleic acid in CO-FA. ^13^C NMR analysis of **10** confirmed the hybridization of curucumenone in CZ1 and ricinoleic acid in CO-FA together with incorporation of benzylamine and cyclohexyl isocyanide (Fig. 6): the signal of the carbonyl ketone of curcumenone (210 ppm) disappeared, and correspondingly, a peak for the newly constructed tetra-substituted carbon of **10** was observed at 65 ppm. In addition, both the acid and isonitrile carbons were transformed into amide carbonyl carbons with peaks at approximately 175 ppm. The signals of the other structural motifs of four substrate components were completely retained in the hybrid-type product **10**. Additionally, further separation and purification of the newly appeared spots (compounds) led to the isolation of two plant product-like Ugi adducts **5** (1.26 mg, 2.0%) and **9** (12.3 mg), and three hybridized Ugi adducts **11** (2.97 mg, 2.3%), **12** and **13** (0.73 mg, 0.8%) (Fig. 7). Notably, TLC analysis showed that most components in the CZ1 extract remained unchanged under the present engineering conditions (Fig. 7a,b, and Supplementary Fig. S7). High chemoselectivity ensured facile detection and purification of the products, even from complex mixtures. By contrast, no product was detected by simply stirring a mixture of CZ1 and CO-FA in methanol at room temperature for 7 days (Supplementary Figs. S8d,d′, S9d,d′). This control experiment supported that the isolated products herein did not originate from the extracts themselves, but were indisputably synthesized by the present Ugi-4CR-based engineering process. The conversion efficiency based on the curcumenone content in the CZ1 extract was determined to be 62%, which was comparable to the conversion efficiency of the CZ1 extract (58%, Table 1). This incredibly high conversion efficiency demonstrated the robustness of the proposed engineering strategy based on the Ugi-4CR. Further detailed analysis coupled with high-resolution liquid chromatography of the engineered extracts may identify unexplored products, including those derived from minor components of the plant extracts. To the best of our knowledge, this is the first report on the simultaneous synthesis of natural product hybrids by chemical engineering of natural product extracts.

### Protease inhibition of Ugi adducts derived from plant products

The prepared Ugi adducts have a diamide motif and were therefore subjected to a protease inhibition assay. Protease inhibitors have received increasing attention for their therapeutic potential against respiratory virus infections including severe acute respiratory syndrome coronavirus 2 (SARS-CoV-2)^34,35^. The inhibitory activity of the Ugi adducts was determined in the presence of 6 nM α-chymotrypsin and 100 μM succinyl-Ala-Ala-Pro-Phe-*p*-nitroanilide^36^. Among those tested, the Ugi adducts **7**, **8a**, **8b**, **9**, and **10** exhibited more than 50% inhibitory activity at a concentration of 10 μM, and the corresponding IC_50_s were determined to be 3.1–13.4 μM (Table 3), which were considerably higher (weaker) than that of the reference inhibitor chymostatin A–C (18.5 ± 1.1 nM). By contrast, all the starting materials, such as citronellal, curcumenone, and ricinoleic acid, were inactive against α-chymotrypsin inhibition (Supplementary Table S1). Thus, the proposed engineering method, although preliminary, can be used to create novel candidates for α-chymotrypsin inhibitors. Further structural optimization, such as removal of the benzyl group on the amide nitrogen, construction of a dipeptide motif, and installation of an aromatic amino acid residue at the C-terminal of dipeptides^37^ will improve the inhibitory activities of the Ugi adducts and will be reported in due course. The molecular weights of the synthesized plant product-like molecules were in the range of 492.70–1011.57 g mol^−1^, which corresponds to the category of beyond the rule of five (bRo5)^38^, or middle-molecules. Therefore, the present chemical hybridization method could be used to construct a small library of middle-molecules mimicking natural products, which is useful for the discovery of peptidic modulators of protein–protein interactions^39^. Ongoing biological evaluations of the present molecules will unveil their specific pharmaceutical potential.

**Table 3:** α-Chymotrypsin inhibitory activity of Ugi adducts derived from plant products.

Compound	% Inhibition (10 μM)^a^	IC_50_ (μM)^a^
**2**	9.2 ± 1.6	–
**4**	3.1 ± 0.9	–
**5**	4.2 ± 1.7	–
**7**	90.4 ± 1.1	3.1 ± 0.1
**8a**	57.8 ± 2.4	7.3 ± 0.6
**8b**	48.5 ± 2.6	13.4 ± 1.3
**9**	55.9 ± 1.9	7.9 ± 0.5
**10**	53.1 ± 1.3	7.7 ± 0.6
Chymostatin		18.5 ± 1.1^b^

^a^Data represent the mean ± SE of at least three independent experiments. ^b^nM.

## Conclusion

In conclusion, this study achieved the design and synthesis of 13 natural product-like Ugi adducts by the racemic Ugi-4CR-based engineering of plant extracts. In particular, engineering a mixture of the methanol extract of *C. zedoaria* and castor oil fatty acids successfully demonstrated a novel synthetic route to a series of natural product hybrids **10**–**13**, whereby otherwise unencountered naturally occurring molecules of different origins were chemically hybridized in complex media. The step- and atom-economical Ugi-4CR performed under mild reaction conditions realized highly chemoselective, reproducible, and efficient engineering of plant extracts regardless of their chemical composition. The highly chemoselective manipulation of plant extracts enabled the facile identification of products from complex mixtures by basic TLC analysis, and thereby their rapid isolation by a combination of conventional silica gel column chromatography and preparative TLC, and the solid characterization of their chemical structures by a set of spectral analyses. Since the proposed chemical hybridization could be performed without isolating the individual coupling components from the extracts, the unexplored, minor, and unstable chemicals buried in the plants were directly employed to synthesize unnatural molecules, and concomitantly the presence of these unidentified molecules in plants was unveiled. In principle, the use of extracts containing naturally occurring primary amines and isocyanides^40^, although scarce^24,25^, would deliver triple- and quadruple-hybridized molecules mimicking natural products. Overall, the Ugi-4CR, which cannot be exploited by plants, was effectively utilized to develop an unexplored chemical space. The present synthesis of natural product hybrids in complex media has several advantages over traditional stepwise synthetic methods, and thus opens up new avenues in the studies of natural product chemistry, synthetic chemistry, and medicinal chemistry.

## Methods

### General experimental information

Analytical thin layer chromatography (TLC) was performed using TLC Silica gel 60 F_254_ (Merck) and visualized by UV light at 254 and 345 nm and stained with an acidic solution of *p*-anisaldehyde (concentrated H_2_SO_4_ in EtOH). Silica gel column chromatography was performed using Silica gel 60 (spherical) 40–50 μm (Kanto). Preparative TLC was performed using PTC Silica gel 60 F_254_, 0.5 mm (Merck). The NMR spectra were recorded at room temperature on JEOL ECA 600 or ECS 400 instruments with tetramethylsilane (TMS) as an internal standard. The ^1^H NMR data are presented as follows. Chemical shift (δ in ppm), multiplicity, integration, and coupling constant *J* (in Hz and rounded to 0.1 Hz). Splitting patterns are abbreviated as follows: singlet (s), doublet (d), triplet (t), quintet (quint), multiplet (m), and broad (br), or a combination of them. The ^13^C NMR data are reported in terms of chemical shift (δ in ppm and rounded to 0.1 Hz). The IR spectra were recorded using a JASCO FT/IR 4700 with substance as a neat film on KBr plate or a pellet in a mixture with KBr, and described as wave numbers (cm^−1^). The TOFMS spectra were obtained from a Bruker Daltonics micrOTOF-KSIfocus spectrometer. The optical rotation was measured with an Anton Paar polarimeter MCP300 in a 100 mm-long 2 mL cell at 589 nm at 26 °C. The fluorescence spectra were measured on a JASCO FP-8500 spectroflurometer equipped with a quartz cuvette of 10 mm path length at 25 °C. With 342 nm excitation, the emission spectra were recorded from 350 to 600 nm. Three spectra were accumulated and averaged, the blank spectra were subtracted. Absorbance was measured with a JASCO V-660 spectrometer at 25 °C. Dimethyl sulfoxide (DMSO) for spectrophotometry was purchased from TCI and used as received. Milli-Q water was used throughout. Other reagents were purchased from TCI, Nacalai, Fujifilm Wako, Sigma-Aldrich, and Kanto, and used as received.

### Plants

The pericarp of *Zanthoxylum piperitum* (Lot no. 004819001) and the dried rhizome powder of *Crucuma phaeocaulis* (Lot no. 048920003) were purchased from Tochimoto Tenkaido (Japan). The dried rhizome powder of *Curcuma zedoaria* (Lot no. IW29207, K9Q9207) was purchased from Uchida Wakanyaku (Japan). The dried rhizome powders of *C. zedoaria* (Lot no. GG12201) and *Curcuma longa* were kindly gifted from Keimeido (Japan). Castor oil fatty acids (Lot no. 1908071) was kindly gifted from Itoh Oil Chemicals (Japan). The formal identification of the plant materials used in this study was performed by their suppliers. Their voucher specimens have not been deposited in a public herbarium. Other information about the plant materials is available from the corresponding author upon request. All the plant experiments were in compliance with relevant institutional, national, and international guidelines and legislation.

### Quantitative determination of carbonyl content of plant extracts

The carbonyl content of plant extracts was determined according to the previous procedure^26^ with a slight modification. For the dinitrophenylhydrazine (DNPH) solution, 0.2 g of the solid DNPH was dissolved in methanol/H_2_O/concentrated HCl (25:23:2, 100 mL) just before use. For the KOH solution, 5 g of KOH was dissolved in methanol/H_2_O (4:1, 50 mL) and the solution was stored at 4 °C. To a solution of plant extract (2 mg mL^−1^, 25 μL) in methanol (250 μL), the DNPH solution (225 μL) was added and the resulting mixture was incubated at 50 °C for 50 min. Then, KOH solution (750 μL) was added, and the resulting mixture was stand at room temperature for 10 min. Then, the absorbance was measured against 480 nm. The carbonyl content of plant extracts was described as mg menthone equivalent/mg extract.

### General synthetic procedure for the Ugi reaction

To a solution of aldehyde or ketone (0.5 mmol, 1.0 eq.) in methanol (0.5 M), amine (0.5 mmol, 1.0 eq.) was added at room temperature and the resulting mixture was stirred at room temperature for 1 h. Then, carboxylic acid (0.5 mmol, 1.0 eq.) was added and the resulting solution was cooled to 0 °C. Then, isocyanide (0.5 mmol, 1.0 eq.) was added at 0 °C and the reaction mixture was stirred at 0 °C for 1 h and then at room temperature for 7 days. After the resulting solution was concentrated in vacuo, the crude mixture was purified by silica gel column chromatography.

### α-Chymotrypsin inhibitory assay

Bovine pancreatic α-chymotrypsin was purchased from Sigma-Aldrich (C4129) and used as received. Enzyme solutions were kept on ice during the experiments. The molecular mass of α-chymotrypsin was taken as 25 kDa. *N*-Succinyl-l-alanine-l-alanine-l-proline-l-phenylalanine-*p*-nitroanilide (SAAPFpNA) was purchased from Sigma-Aldrich (S7388), prepared as a 10 mM stock solution in DMSO, and stored at − 30 °C. A microbial chymostatin, a mixture of chymostatin A–C, was purchased from Peptide Institute (4063) and used as a positive control. DMSO for biochemical research was purchased from Nacalai and used as received. Other reagents were purchased from Nacalai and used as received. The concentration–response curves were plotted and IC_50_ values were calculated by GraphPad Prism 8 software using a sigmoidal concentration–response curve analysis with variable slope. Absorbance was measured with a JASCO V-660 spectrometer at 25 °C. According to the reported procedure^36^, the assay was performed in Tris–HCl buffer (80 mM, pH 7.8) containing α-chymotrypsin (6 nM, final concentration) and CaCl_2_ (10 mM, final concentration). After 30 min of incubation with the test compound at 25 °C, the enzymatic reaction was initiated by adding SAAPFpNA (100 μM, final concentration) to give a final volume of 700 μL. Reaction progress was monitored by absorbance at 410 nm at 25 °C for 5 min. The final concentration of DMSO was 3% (v/v).

## Supplementary Information

Supplementary Information.

## Data Availability

Supplementary figures and tables as mentioned in the text, synthetic details, and NMR spectra are available in the supplementary information file. Other information needed is available from the corresponding author upon request.
