# Supplementary material for: Synthesis of natural product hybrids by the Ugi reaction in complex media containing plant extracts
Source: Sci Rep. 2022 Sep 16;12:15568. doi: 10.1038/s41598-022-19579-6 (PMC9481608; doi:10.1038/s41598-022-19579-6)
Supplement: Supplementary file 1 — Supplementary Information. [file 41598_2022_19579_MOESM1_ESM.docx]

**Supporting information**

**Synthesis of natural product hybrids by the Ugi reaction in complex media containing plant extracts**

Keisuke Tomohara^*,1^, Nao Ohashi^2^, Tatsuya Uchida^1,2,3^, and Takeru Nose^1,2^

^1^Faculty of Arts and Science, Kyushu University, 744 Motooka, Nishi-ku, Fukuoka 819-0395, Japan

^2^Graduate School of Science, Kyushu University, 744 Motooka, Nishi-ku, Fukuoka 819-0395, Japan

^3^International Institute for Carbon-Neutral Energy Research, Kyushu University, 744 Motooka, Nishi-ku, Fukuoka 819-0395, Japan

**Table of Contents**

1. Supplementary Figures S1–S9 3

2. Supplementary Tables S1–S2 11

3. Synthetic details & Supplementary Figures S10–S19 13

4. References 53

5. Supplementary Figures S20–S100 (NMR spectra) 54

**1. Supplementary Figures S1–S9**

**Figure S1.** Substrate scope of the Ugi-4CR.


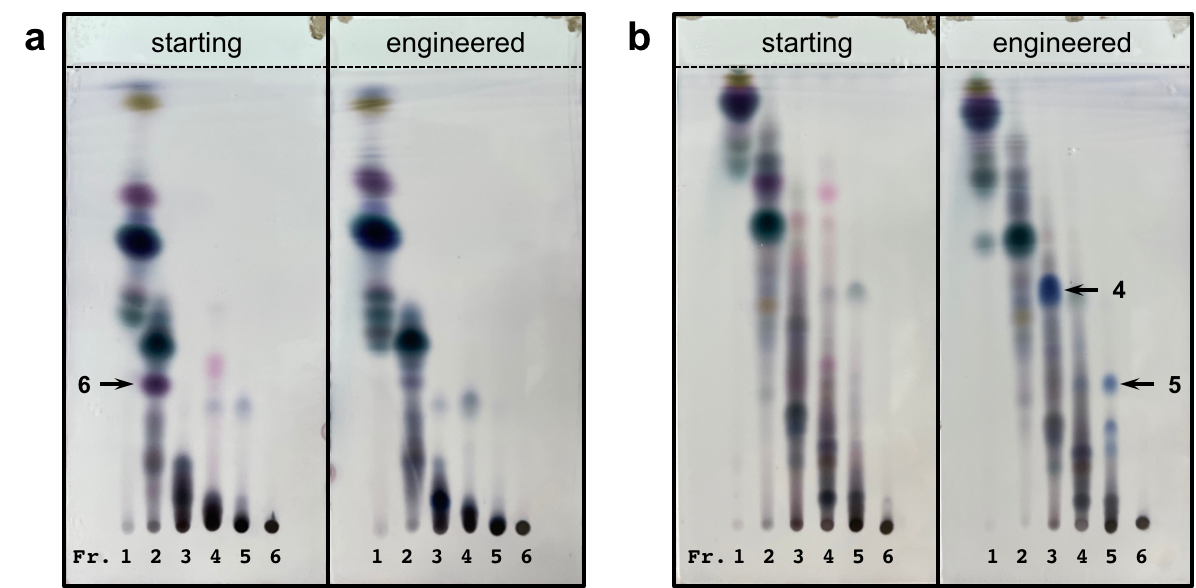


**Figure S2.** TLC analysis of the fractionated starting and engineered extracts of *Curcuma zedoaria* (CZ1). The TLCs were developed with (a) hexane/ethyl acetate (4:1) or with (b) 1,2-dichloroethane/ethyl acetate (19:6), and then visualized by staining with an acidic solution of *p*-anisaldehyde*.* The upper hash line indicates the solvent front.


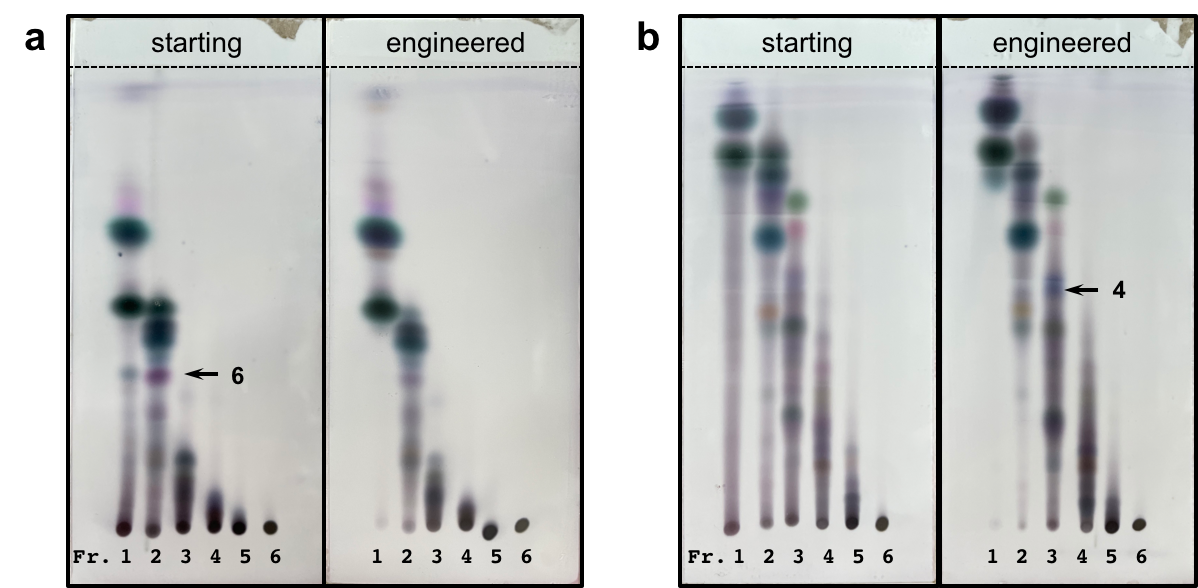


**Figure S3.** TLC analysis of the fractionated starting and engineered extracts of *Curcuma zedoaria* (CZ2). The TLCs were developed with (a) hexane/ethyl acetate (4:1) or with (b) 1,2-dichloroethane/ethyl acetate (19:6), and then visualized by staining with an acidic solution of *p*-anisaldehyde. The upper hash line indicates the solvent front.


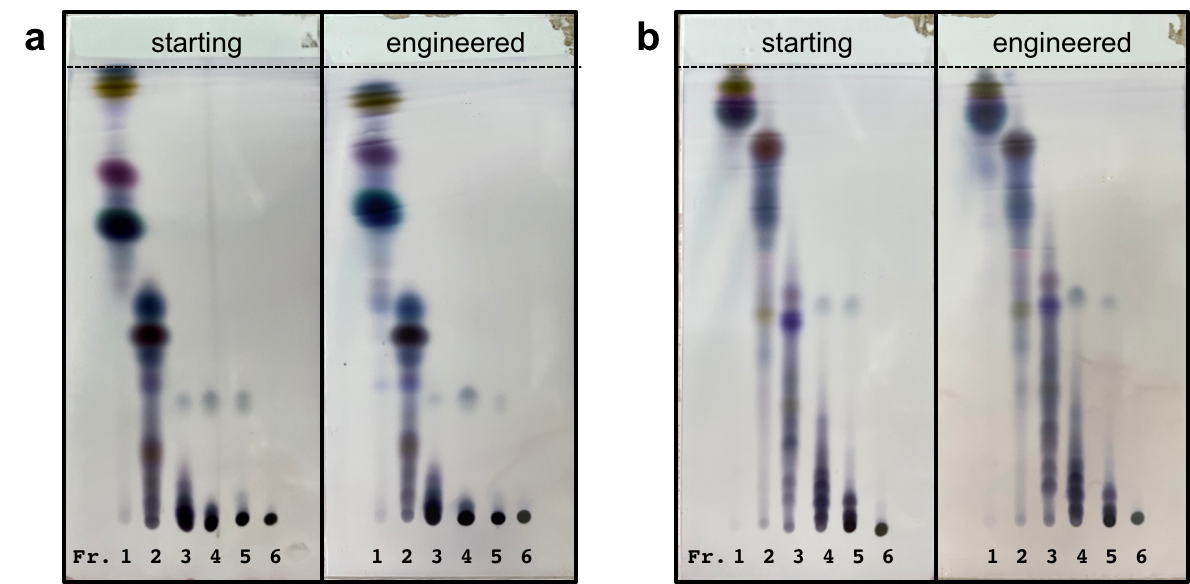


**Figure S4.** TLC analysis of the fractionated starting and engineered extracts of *Curcuma phaeocaulis* (CP1). The TLCs were developed with (a) hexane/ethyl acetate (4:1) or with (b) 1,2-dichloroethane/ethyl acetate (19:6), and then visualized by staining with an acidic solution of *p*-anisaldehyde. The upper hash line indicates the solvent front.


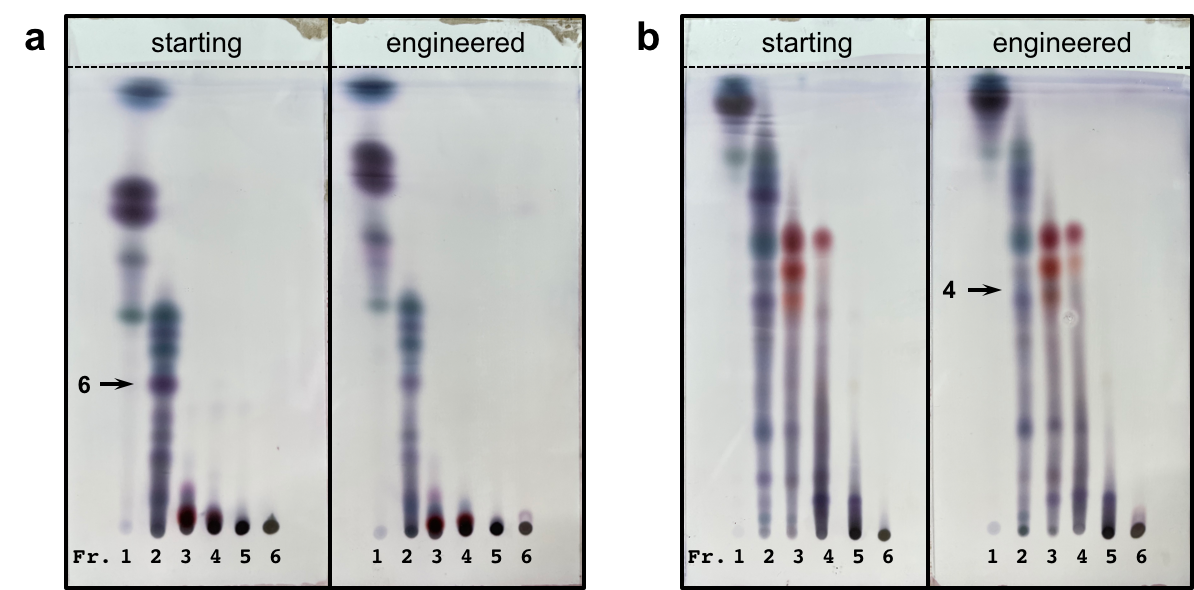


**Figure S5.** TLC analysis of the fractionated starting and engineered extracts of *Curcuma longa* (CL1). The TLCs were developed with (a) hexane/ethyl acetate (4:1) or with (b) 1,2-dichloroethane/ethyl acetate (19:6), and then visualized by staining with an acidic solution of *p*-anisaldehyde*.* The upper hash line indicates the solvent front.

**
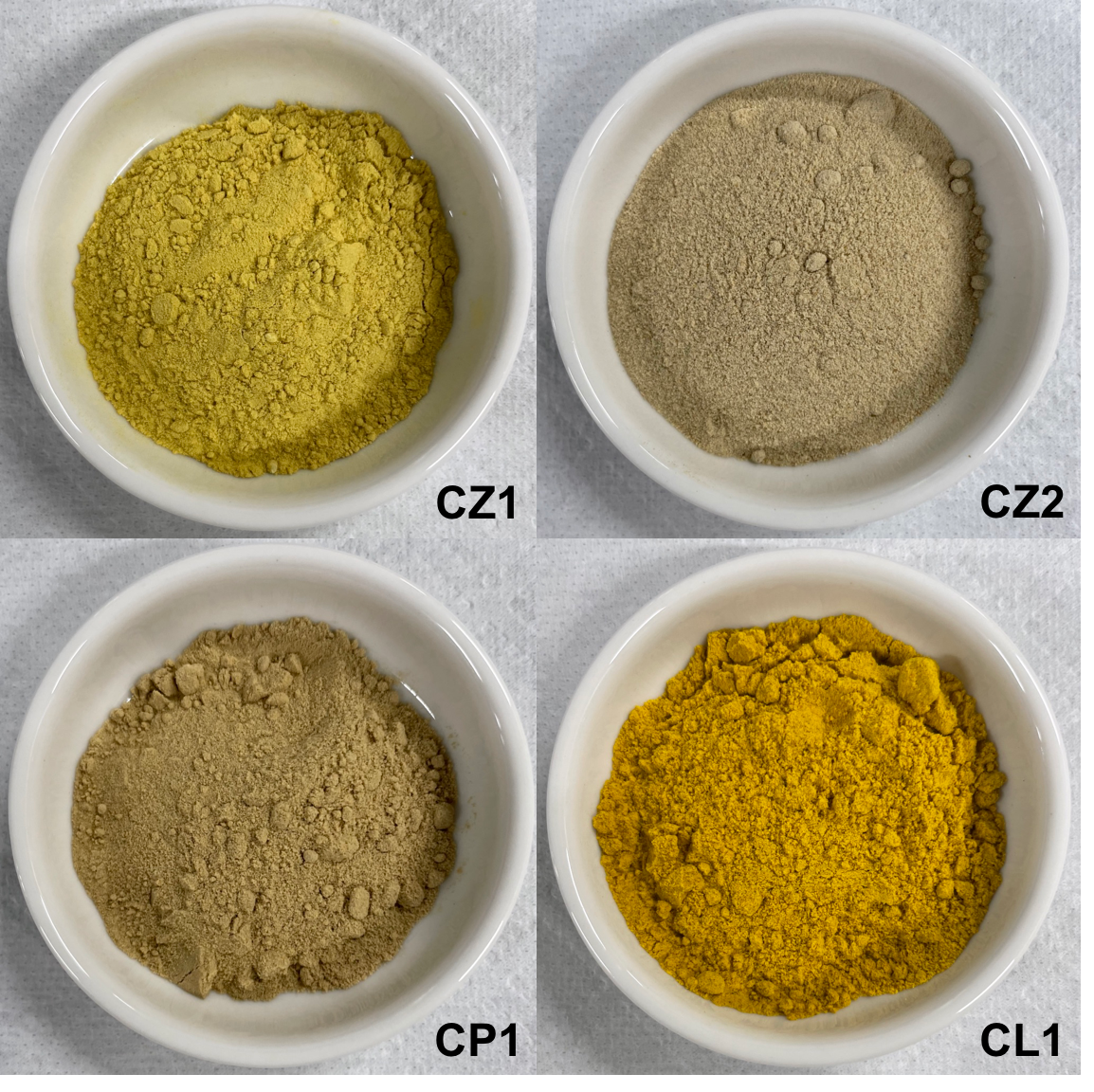
**

**Figure S6.** Pictures of dried rhizome powders of *Curcuma zedoaria* (CZ1 and CZ2), *Curcuma* *paeocaulis* (CP1), and *Curcuma longa* (CL1).


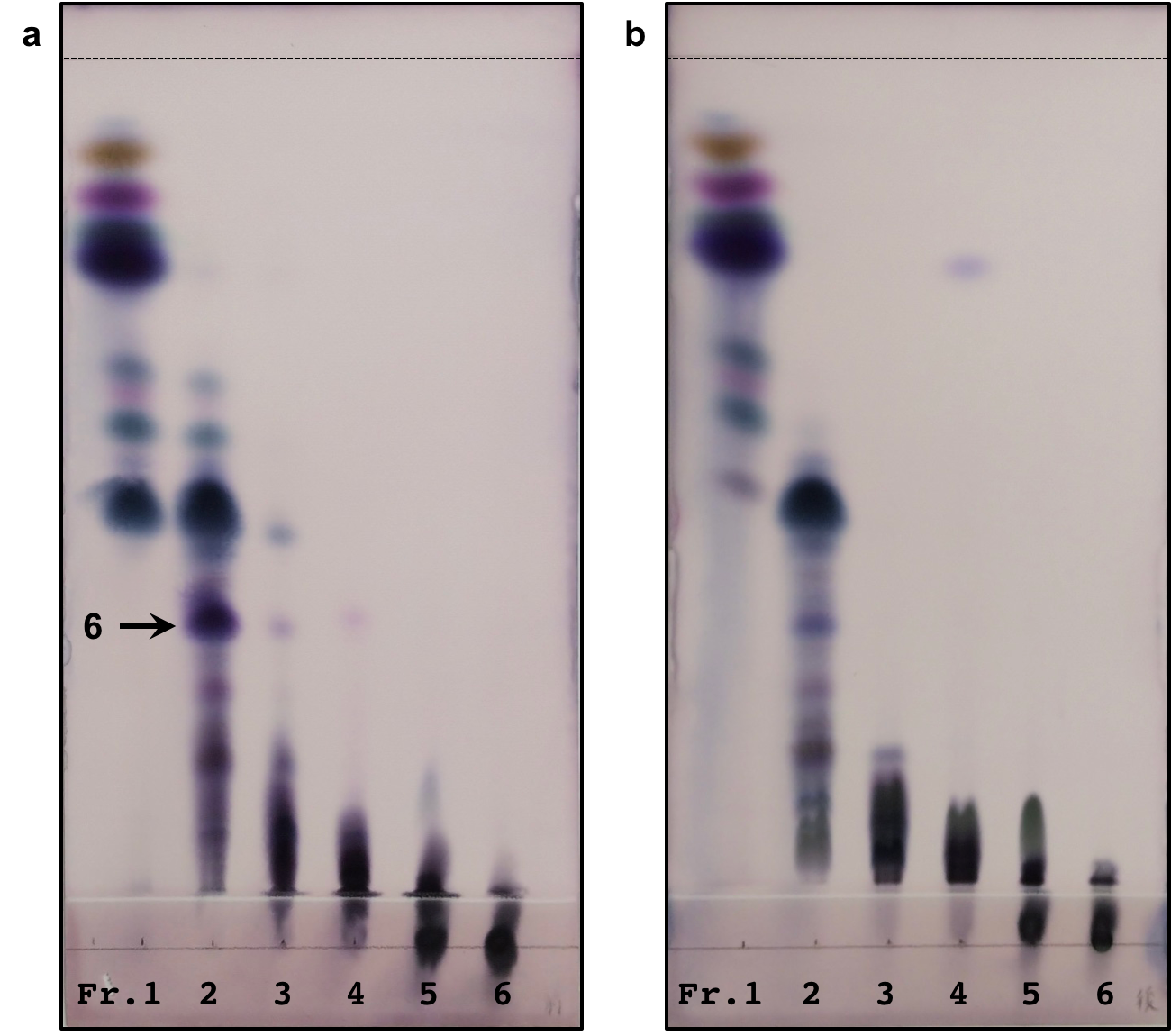


**Figure S7-1.** TLC analysis of the fractionated extract of *Curcuma zedoaria* (CZ1) (a) and the fractionated reaction mixture (b). The TLCs were developed with hexane/ethyl acetate (4:1), and then visualized by staining with an acidic solution of *p*-anisaldehyde. The upper hash line indicates the solvent front.


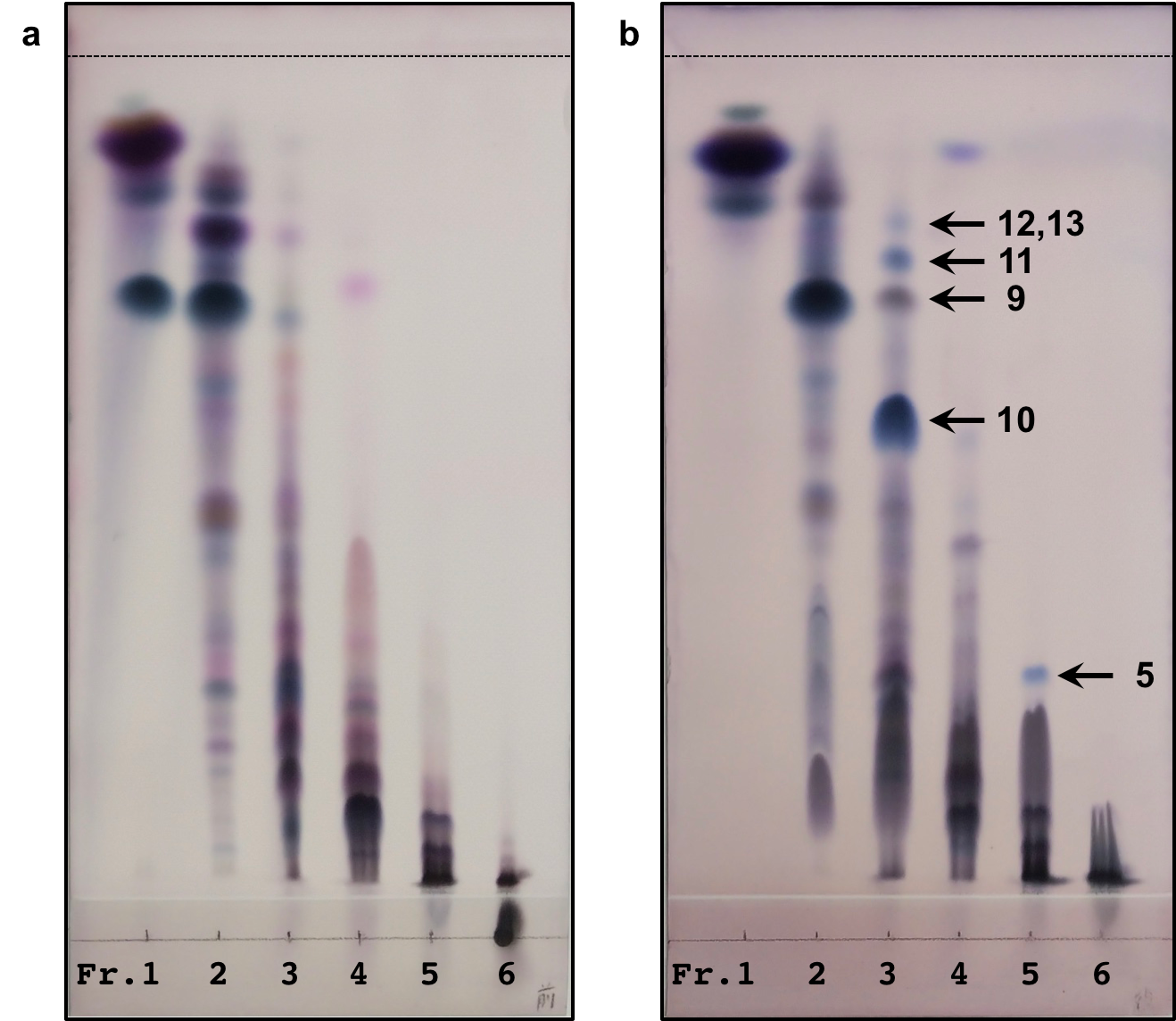


**Figure S7-2.** (a) TLC of the fractionated starting extract of *C. zedoaria* (CZ1). (b) TLC of the fractionated engineered mixture. Both TLCs were developed with CHCl_3_/ethyl acetate (19:6) and then visualized by staining with an acidic solution of *p*-anisaldehyde. The upper hash line indicates the solvent front.

**
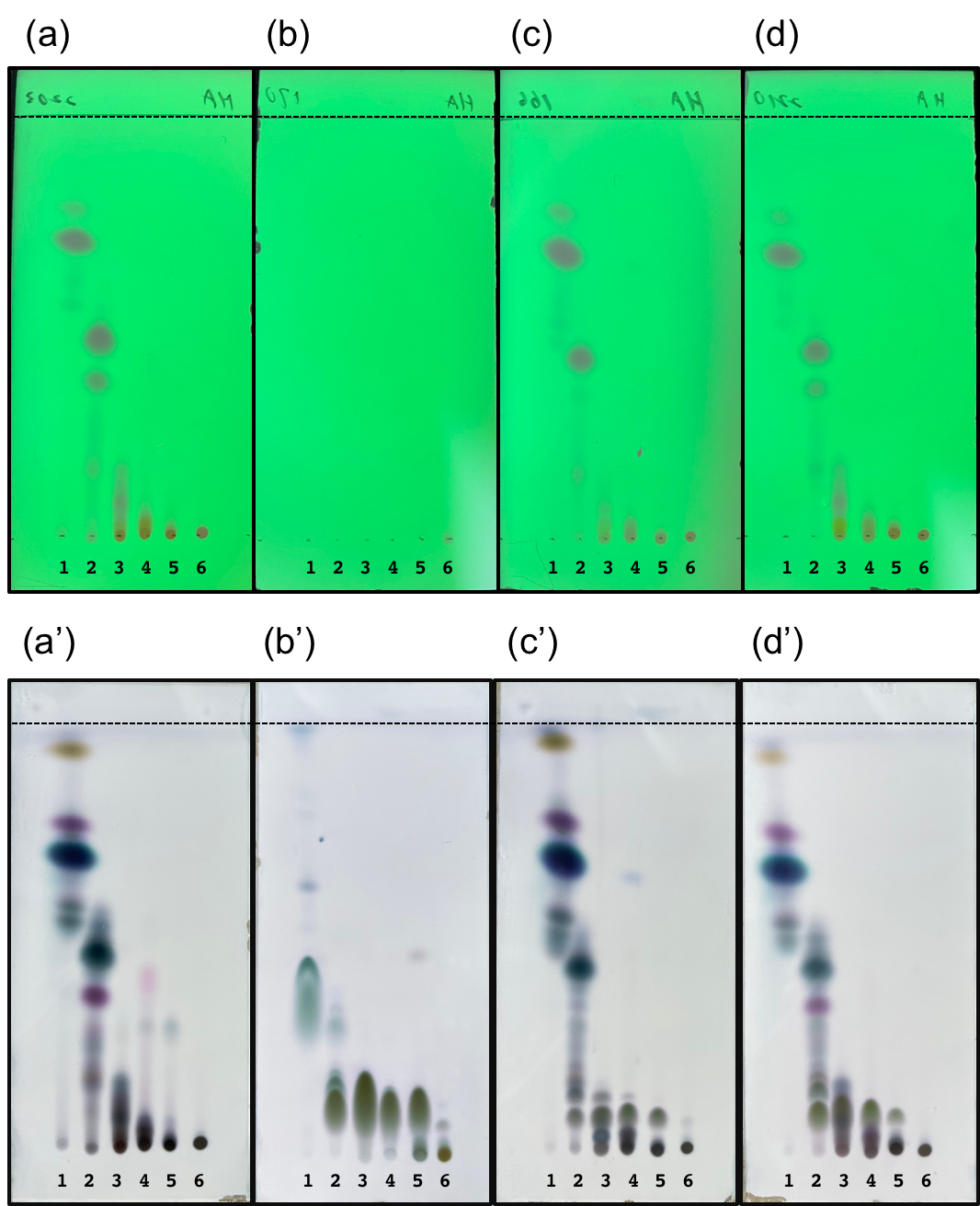
**

**Figure S8.** TLC analysis of the fractionated extract of *C. zedoaria* (CZ1) (a and a’), the fractionated castor oil fatty acids (CO-FA) (b and b’), the fractionated reaction mixture (c and c’), and the control mixture (d and d’). The TLCs were developed with hexane/ethyl acetate (4:1), and then visualized by UV light at 254 nm (a–d) or staining with an acidic solution of *p*-anisaldehyde (a’–d’). The upper hash line indicates the solvent front.

**
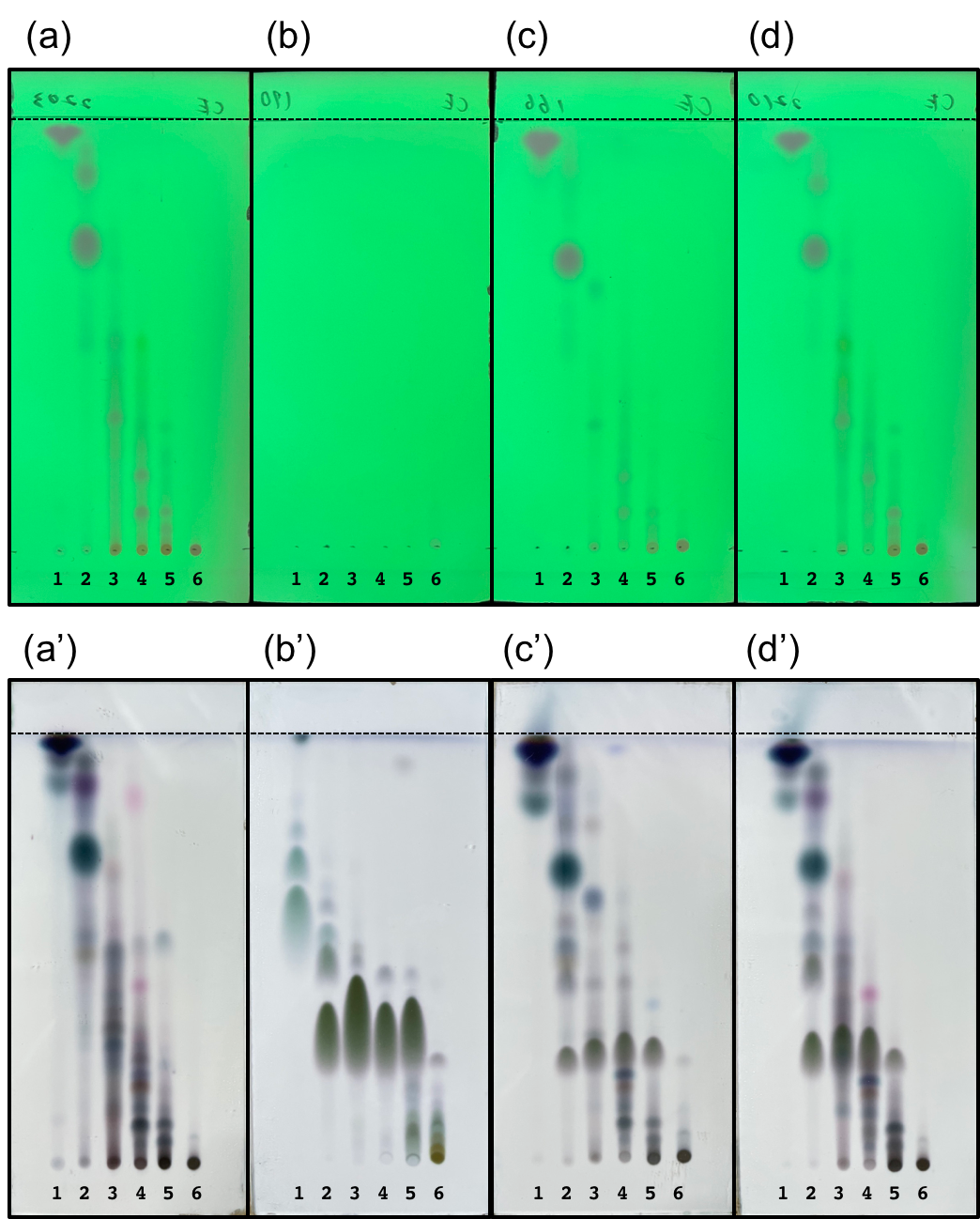
**

**Figure S9.** TLC analysis of the fractionated extract of *C. zedoaria* (a and a’), the fractionated castor oil fatty acid (b and b’), the fractionated reaction mixture (c and c’), and the control mixture (d and d’). The TLCs were developed with CHCl_3_/ethyl acetate (19:6), and then visualized by UV light at 254 nm (a–d) or by staining with an acidic solution of *p*-anisaldehyde (a’–d’). The upper hash line indicates the solvent front.

**2. Supplementary Tables S1–S2**

**Table S1. α-Chymotrypsin inhibitory activity of the starting materials**

| Compound | % Inhibition (10 μM)^a^ |
| --- | --- |
| Benzylamine | 7.0 ± 1.4 |
| 1-Aminopyrene | 5.6 ± 1.0 |
| TosMIC | 0.5 ± 0.5 |
| Cyclohexyl isocyanide | 1.4 ± 1.1 |
| (±)-Citronellal (**3**) | 5.9 ± 1.1 |
| Curcumenone (**6**) | 7.8 ± 1.2 |
| (*R*)-Ricinoleic acid | 3.0 ± 0.9 |

^a^Data represent the mean ± SE of at least three independent experiments.

**Table S2. α-Chymotrypsin inhibitory activity of the Ugi adducts**

| Compound | % Inhibition (10 μM)^a^ | IC_50_ (μM)^a^ |
| --- | --- | --- |
| **1a** | 4.7 ± 1.1 | - |
| **1b** | 9.2 ± 2.0 | - |
| **1c** | 4.4 ± 2.0 | - |
| **1d** | 8.9 ± 2.1 | - |
| **1e** | 0.2 ± 0.8 | - |
| **1f** | 7.1 ± 1.0 | - |
| **1g** | 83.7 ± 1.8 | 4.5 ± 0.3 |
| **1h** | 2.4 ± 1.6 | - |
| **1i** | 3.8 ± 0.4 | - |
| **1j** | 62.3 ± 0.2 | 9.2 ± 0.8 |

^a^Data represent the mean ± SE of at least three independent experiments.

**3. Synthetic Details & Supplementary Figures S10–S19**

**General synthetic procedure for the Ugi reaction**

To a solution of aldehyde or ketone (0.5 mmol, 1.0 eq.) in methanol (0.5 M), amine (0.5 mmol, 1.0 eq.) was added at room temperature and the resulting mixture was stirred at room temperature for 1 hour. Then, carboxylic acid (0.5 mmol, 1.0 eq.) was added and the resulting solution was cooled to 0 °C. Then, isocyanide (0.5 mmol, 1.0 eq.) was added at 0 °C and the reaction mixture was stirred at 0 °C for 1 hour and then at room temperature for 7 days. After the resulting solution was concentrated *in vacuo*, the crude mixture was purified by silica gel column chromatography.

**Synthesis of 1a**

According to the general procedure, benzaldehyde (53.0 mg, 0.5 mmol), benzylamine (53.7 mg, 0.5 mmol), chloroacetic acid (47.3 mg, 0.5 mmol), and *p*-toluenesulfonylmethyl isocyanide (TosMIC) (97.7 mg, 0.5 mmol) were converted into the corresponding Ugi adduct **1a** in 62% yield (151 mg) as a colorless amorphous solid after silica gel column chromatography (hexane/ethyl acetate = 2:1 to 1:1).

**1a**: *N*-Benzyl-2-chloro-*N*-(2-oxo-1-phenyl-2-((tosylmethyl)amino)ethyl)acetamide

**^1^H NMR** (600 MHz, CDCl_3_): δ 2.43 (s, 3H), 3.90 (d, 1H, *J* = 13.5 Hz), 4.00 (d, 1H, *J* =13.5 Hz), 4.48 (d, 1H, *J* = 17.1 Hz), 4.63 (d, 1H, *J* = 17.1 Hz), 4.66–4.72 (m, 2H), 5.96 (br s, 1H), 6.81–6.84 (m, 1H), 6.89–6.90 (m, 2H), 7.16–7.30 (m, 10H), 7.70 (d, 2H, *J* = 8.4 Hz).

**^13^C NMR** (151 MHz, CDCl_3_): δ 21.8 (CH_3_), 42.4 (CH_2_), 49.5 (CH_2_), 60.5 (CH_2_), 62.7 (CH), 126.0 (CH), 127.4 (CH), 128.7 (CH), 128.9 (CH), 128.9 (CH), 129.1 (CH), 130.0 (CH), 130.0 (CH), 133.1 (C), 134.0 (C), 136.6 (C), 145.2 (C), 168.6 (C), 169.4 (C).

**IR** (KBr): cm^-1^ 3314, 3061, 3032, 2929, 1699, 1656, 1536, 1454, 1410, 1321, 1143, 754, 734, 699, 567, 515.

**HRMS** (ESI-TOF): *m/z* calcd for C_25_H_25_ClN_2_NaO_4_S ([M + Na]^+^): 507.1116; found: 507.1116.

**Synthesis of 1b**

According to the general procedure, benzaldehyde (53.0 mg, 0.5 mmol), benzylamine (53.7 mg, 0.5 mmol), chloroacetic acid (47.5 mg, 0.5 mmol), and cyclohexyl isocyanide (54.6 mg, 0.5 mmol) were converted into the corresponding Ugi adduct **1b**^1^ in 89 % yield (178 mg) as a colorless solid after silica gel column chromatography (hexane/ethyl acetate = 3:1).

**1b**: *N*-Benzyl-2-chloro-*N*-(2-(cyclohexylamino)-2-oxo-1-phenylethyl)acetamide

**^1^H NMR** (600 MHz, CDCl_3_): δ 1.04–1.15 (m, 3H), 1.30–1.36 (m, 2H), 1.57–1.67 (m, 3H), 1.88–1.93 (m, 2H), 3.80 (s, 1H), 3.93 (d, 1H, *J* = 12.6 Hz), 4.04 (d, 1H, *J* = 12.6 Hz), 4.59 (d, 1H, *J* = 17.7 Hz), 4.78 (d, 1H, *J* = 17.7 Hz), 5.55 (br s, 1H), 5.88 (s, 1H), 7.04 (d, 2H, *J* = 6.6 Hz), 7.16–7.22 (m, 3H), 7.29–7.39 (m, 5H).

**IR** (KBr): cm^-1^ 3264, 3087, 2932, 2855, 1664, 1646, 1563, 1453, 1414, 698.

**HRMS** (ESI-TOF): *m/z* calcd for C_23_H_27_ClN_2_NaO_2_ ([M + Na]^+^): 421.1653; found: 421.1651.

**Synthesis of 1c**

According to the general procedure, isobutylaldehyde (36.0 mg, 0.5 mmol), benzylamine (53.7 mg, 0.5 mmol), chloroacetic acid (49.6 mg, 0.5 mmol), and cyclohexyl isocyanide (54.6 mg, 0.5 mmol) were converted into the corresponding Ugi adduct **1c** in 73 % yield (133 mg) a colorless amorphous solid after silica gel column chromatography (hexane/ethyl acetate = 4:1 to 2:1).

**1c**: 2-(*N*-Benzyl-2-chloroacetamido)-*N*-cyclohexyl-3-methylbutanamide

**^1^H NMR** (600 MHz, CDCl_3_): δ 0.90 (d, 3H, *J* = 6.6 Hz), 0.96 (d, 3H, *J* = 6.6 Hz), 1.13–1.21 (m, 3H), 1.30–1.37 (m, 2H), 1.58–1.61 (m, 1H), 1.67–1.72 (m, 2H), 1.82–1.88 (m, 2H), 2.40–2.47 (m, 1H), 3.62–3.68 (m, 1H), 3.84 (d, 1H, *J* = 12.6 Hz), 3.95 (d, 1H, *J* = 12.6 Hz), 4.43 (br d, 1H, *J* = 10.2 Hz), 4.65 (d, 1H, *J* = 17.7 Hz), 4.91 (d, 1H, *J* = 17.7 Hz), 6.28 (br s, 1H), 7.14 (d, 2H, *J* = 7.8 Hz), 7.26 (t, 1H, *J* = 7.8 Hz), 7.31 (t, 2H, *J* = 7.8 Hz).

**^13^C NMR** (151 MHz, CDCl_3_): δ 18.6 (CH_3_), 19.5 (CH_3_), 24.7 (CH_2_), 24.7 (CH_2_), 25.4 (CH_2_), 27.7 (CH), 32.5 (CH_2_), 32.8 (CH_2_), 42.0 (CH_2_), 48.1 (CH), 48.1 (CH_2_), 64.9 (CH), 125.8 (CH), 127.4 (CH), 128.8 (CH), 137.1 (C), 168.2 (C), 168.8 (C).

**IR** (KBr): cm^-1^ 3319, 3068, 3032, 2932, 2855, 1642, 1545, 1452, 1415, 1363, 755, 729, 696, 673.

**HRMS** (ESI-TOF): *m/z* calcd for C_20_H_29_ClN_2_NaO_2_ ([M + Na]^+^): 387.1810; found: 387.1810.

**Synthesis of 1d**

According to the general procedure, β-cyclocitral (76.1 mg, 0.5 mmol), benzylamine (53.6 mg, 0.5 mmol), chloroacetic acid (47.8 mg, 0.5 mmol), and cyclohexyl isocyanide (54.6 mg, 0.5 mmol) were converted into the corresponding Ugi adduct **1d** in 45 % yield (99.8 mg) a colorless amorphous solid after silica gel column chromatography (hexane/ethyl acetate = 5:1 to 4:1).

**1d**: *N*-Benzyl-2-chloro-*N*-(2-(cyclohexylamino)-2-oxo-1-(2,6,6-trimethylcyclohex-1-en-1-yl)ethyl)acetamide

**^1^H NMR** (400 MHz, CDCl_3_): δ 0.95 (s, 3H), 1.01–1.20 (m, 3H), 1.20 (s, 3H), 1.31–1.50 (m, 3H), 1.40 (s. 3H), 1.57–1.69 (m, 6H), 1.83–1.94 (m, 2H), 1.99–2.07 (m, 2H), 3.77–3.84 (m, 1H), 3.89 (d, 1H, *J* = 12.4 Hz), 3.96 (d, 1H, *J* = 12.4 Hz), 4.54 (d, 1H, *J* = 18.2 Hz), 4.65 (d, 1H, *J* = 18.2 Hz), 5.23 (br d, 1H, *J* = 8.4 Hz), 5.62 (s, 1H), 7.26 (d, 1H, *J* = 7.2 Hz), 7.37 (t, 2H, *J* = 7.2 Hz), 7.65 (d, 2H, *J* = 7.2 Hz).

**^13^C NMR** (151 MHz, CDCl_3_): δ 18.8 (CH_2_), 22.4 (CH_3_), 24.6 (CH_2_), 24.7 (CH_2_), 25.5 (CH_2_), 27.7 (CH_3_), 28.7 (CH_3_), 33.1 (CH_2_), 33.1 (CH_2_), 33.5 (CH_2_), 35.9 (C), 39.0 (CH_2_), 42.1 (CH_2_), 48.1 (CH_2_), 48.1 (CH), 58.9 (CH), 126.4 (CH), 127.2 (CH), 128.9 (CH), 132.6 (C), 138.2 (C), 140.7 (C), 168.3 (C), 169.9 (C).

**IR** (KBr): cm^-1^ 3411, 3051, 2932, 2855, 1678, 1666, 1509, 1454, 1405, 1379, 802, 732.

**HRMS** (ESI-TOF): *m/z* calcd for C_26_H_37_ClN_2_NaO_2_ ([M + Na]^+^): 467.2436; found: 467.2439.

**Synthesis of 1e**

According to the general procedure, cyclohexanone (42.1 mg, 0.5 mmol), benzylamine (53.6 mg, 0.5 mmol), chloroacetic acid (47.3 mg, 0.5 mmol), and cyclohexyl isocyanide (54.6 mg, 0.5 mmol) were converted into the corresponding Ugi adduct **1e** in 77 % yield (146 mg) a colorless amorphous solid after silica gel column chromatography (hexane/ethyl acetate = 4:1 to 1:1).

**1e**: 1-(*N*-Benzyl-2-chloroacetamido)-*N*-cyclohexylcyclopentane-1-carboxamide

**^1^H NMR** (400 MHz, CDCl_3_): δ 1.12–1.26 (m, 3H), 1.33–1.39 (m, 2H), 1.58–1.70 (m, 7H), 1.87–1.89 (m, 4H), 2.61–2.63 (m, 2H), 3.71–3.76 (m, 1H), 3.94 (s, 2H), 4.75 (s, 2H), 6.44 (br s, 1H), 7.27 (d, 2H, *J* = 7.2 Hz), 7.31 (t, 1H, *J* =7.2 Hz), 7.39 (t, 2H, *J* = 7.2 Hz).

**^13^C NMR** (151 MHz, CDCl_3_): δ 23.4 (CH_2_), 24.8 (CH_2_), 25.6 (CH_2_), 32.9 (CH_2_), 36.1 (CH_2_), 42.7 (CH_2_), 48.4 (CH), 50.5 (CH_2_), 74.2 (C), 125.6 (CH), 127.8 (CH), 129.3 (CH), 138.0 (C), 169.1 (C), 172.2 (C).

**IR** (KBr): cm^-1^ 3332, 2939, 2855, 1657, 1524, 1468, 1411, 793, 739, 704.

**HRMS** (ESI-TOF): *m/z* calcd for C_21_H_29_ClN_2_NaO_2_ ([M + Na]^+^): 399.1810; found: 399.1812.

**Synthesis of 1f**

According to the general procedure, cyclohexanone (42.1 mg, 0.5 mmol), benzylamine (53.6 mg, 0.5 mmol), chloroacetic acid (50.1 mg, 0.5 mmol), and TosMIC (99.4 mg, 0.5 mmol) were converted into the corresponding Ugi adduct **1f** in 48 % yield (110 mg) a colorless amorphous solid after silica gel column chromatography (hexane/ethyl acetate = 2:1 to 1:1).

**1f**: 1-(*N*-Benzyl-2-chloroacetamido)-*N*-(tosylmethyl)cyclopentane-1-carboxamide

**^1^H NMR** (600 MHz, CDCl_3_): δ 1.43–1.50 (m, 2H), 1.53–1.60 (m, 2H), 1.79–1.85 (m, 2H), 2.44 (s, 3H), 2.44–2.47 (m, 2H), 4.00 (s, 2H), 4.65 (s, 2H), 4.70 (d, 2H, *J* = 6.6 Hz), 7.20 (d, 2H, *J* = 6.6 Hz), 7.29–7.35 (m, 4H), 7.39–7.41 (m, 2H), 7.79–7.81 (m, 2H).

**^13^C NMR** (151 MHz, CDCl_3_): δ 21.7 (CH_3_), 23.4 (CH_2_), 35.9 (CH_2_), 42.9 (CH_2_), 50.5 (CH_2_), 60.9 (CH_2_), 73.9 (C), 125.5 (CH), 127.9 (CH), 128.8 (CH), 129.3 (CH), 129.9 (CH), 134.5 (C), 137.3 (C), 145.3 (C), 169.1 (C), 173.2 (C).

**IR** (KBr): cm^-1^ 3374, 3008, 2954, 2873, 1667, 1509, 1452, 1412, 1289, 1142, 815, 799, 761, 745.

**HRMS** (ESI-TOF): *m/z* calcd for C_23_H_27_ClN_2_NaO_4_S ([M + Na]^+^): 485.1272; found: 485.1271.

**Synthesis of 1g**

A solution of benzaldehyde (53.0 mg, 0.5 mmol) and 1-aminopyrene (109 mg, 0.5 mmol) in methanol (5 mL, 0.1 M) was stirred at room temperature for 1 h, and then chloroacetic acid (47.3 mg, 0.5 mmol) was added to the mixture. Then, the resulting mixture was cooled to 5 °C and cyclohexyl isocyanide (54.6 mg, 0.5 mmol) was added. The reaction mixture was stirred at 5 °C for 1 h and then at room temperature for 7 days. The precipitates were collected by filtration and the collected solid was washed with cold hexane to afford the corresponding Ugi adduct **1g** in 93 % yield (236 mg) as a beige amorphous solid. The title compound exists as a mixture of two rotamers in a 7:3 ratio at room temperature in CDCl_3_.

**1g**: 2-Chloro-*N*-(2-(cyclohexylamino)-2-oxo-1-phenylethyl)-*N*-(pyren-1-yl)acetamide

**^1^H NMR** (600 MHz, CDCl_3_): δ 1.05–1.21 (m, 3H), 1.31–1.42 (m, 2H), 1.57–1.71 (m, 3H), 1.89–2.02 (m, 2H), 3.69–3.77 (m, 2H), 3.85–3.96 (m, 1H), 5.57 (s, 0.7H), 5.60 (br d, 0.3H, *J* = 7.8 Hz), 5.74 (br d, 0.7H, *J* = 7.8 Hz), 5.97 (s, 0.3H), 6.97–6.98 (m, 0.6H), 7.13–7.22 (m, 3H), 7.38 (d, 1.4H, *J* = 7.2 Hz), 7.79 (d, 0.7H, *J* = 7.8 Hz), 8.00–8.26 (m, 7.6H), 8.41 (d, 0.7H, *J* = 9.0 Hz).

**^13^C NMR** (151 MHz, CDCl_3_): δ 24.9 (CH_2_), 24.9 (CH_2_), 25.6 (CH_2_), 32.9 (CH_2_), 33.0 (CH_2_), 43.0 and 43.2 (CH_2_, diastereomers), 49.0 and 49.1 (CH, diastereomers), 68.0 and 70.2 (CH, diastereomers), 121.8 and 122.4 (CH, diastereomers), 124.3 and 124.4 (C, diastereomers), 125.1 and 125.3 (CH, diastereomers), 125.2 and 125.3 (C, diastereomers), 129.1 and 129.7 (C, diastereomers), 130.7, 130.9, 131.0, 131.1, 131.6, 131.9, 132.4, 133.7, 133.9, 134.7 (5×C, diastereomers), 126.1, 126.2, 126.6, 126.7, 127.1, 127.1, 127.7, 128.2, 128.5, 128.7, 127.7, 128.8, 128.9, 129.0, 129.3, 129.5 130.4, 130.5 (10×CH, diastereomers), 167.8 and 167.8 (C, diastereomers), 168.0 and 168.3 (C, diastereomers).

**IR** (KBr): cm^-1^ 3285, 3044, 2930, 2853, 1678, 1652, 1549, 1453, 1438, 1409, 848, 701.

**HRMS** (ESI-TOF): *m/z* calcd for C_32_H_29_ClN_2_NaO_2_ ([M + Na]^+^): 531.1810; found: 531.1810.

**Synthesis of 1h**

According to the general procedure, benzaldehyde (52.8 mg, 0.5 mmol), benzylamine (53.6 mg, 0.5 mmol), acetic acid (30.0 mg, 0.5 mmol), and cyclohexyl isocyanide (54.6 mg, 0.5 mmol) were converted into the corresponding Ugi adduct **1h** in 46 % yield (84.7 mg) a colorless amorphous solid after silica gel column chromatography (hexane/ethyl acetate = 2:1 to 1:2).

**1h**: 2-(*N*-Benzylacetamido)-*N*-cyclohexyl-2-phenylacetamide

**^1^H NMR** (600 MHz, CDCl_3_): δ 1.04–1.15 (m, 3H), 1.30–1.37 (m, 2H), 1.56–1.59 (m, 1H), 1.62–1.68 (m, 2H), 1.88–1.91 (m, 2H), 2.08 (s, 3H), 3.78–3.83 (m, 1H), 4.55 (d, 1H, *J* = 17.7 Hz), 4.73 (d, 1H, *J* = 17.7 Hz), 5.56 (br d, 1H, *J* = 6.0 Hz), 5.93 (s, 1H), 7.00 (d, 2H, *J* = 7.2 Hz), 7.12–7.19 (m, 3H), 7.25–7.26 (m, 3H), 7.35–7.37 (m, 2H).

**^13^C NMR** (151 MHz, CDCl_3_): δ 22.5 (CH_3_), 24.8 (CH_2_), 24.8 (CH_2_), 25.5 (CH_2_), 32.7 (CH_2_), 32.7 (CH_2_), 48.6 (CH), 50.7 (CH_2_), 62.4 (CH), 126.1 (CH), 126.8 (CH), 128.3 (CH), 128.4 (CH), 128.7 (CH), 129.7 (CH), 135.4 (C), 137.7 (C), 168.8 (C), 172.6 (C).

**IR** (KBr): cm^-1^ 3277, 3081, 2928, 2854, 1676, 1634, 1561, 1466, 1452, 1374, 723, 706, 693.

**HRMS** (ESI-TOF): *m/z* calcd for C_23_H_28_N_2_NaO_2_ ([M + Na]^+^): 387.2043; found: 387.2043.

**Synthesis of 1i**

According to the general procedure, benzaldehyde (53.0 mg, 0.5 mmol), benzylamine (53.7 mg, 0.5 mmol), cyclohexanecarboxylic acid (64.1 mg, 0.5 mmol), and cyclohexyl isocyanide (54.6 mg, 0.5 mmol) were converted into the corresponding Ugi adduct **1i** in 82 % yield (178 mg) a colorless amorphous solid after silica gel column chromatography (hexane/ethyl acetate = 4:1).

**1i**: *N*-Benzyl-*N*-(2-(cyclohexylamino)-2-oxo-1-phenylethyl)cyclohexanecarboxamide

**^1^H NMR** (600 MHz, CDCl_3_): δ 0.99–1.36 (m, 7H), 1.48–1.92 (m, 13H), 2.39–2.44 (m, 1H), 3.75–3.82 (m, 1H), 4.51 (d, 1H, *J* = 17.4 Hz), 4.75 (d, 1H, *J* = 17.4 Hz), 5.65 (br d, 1H, *J* = 6.6 Hz), 5.89 (s, 1H), 6.99 (d, 2H, *J* = 7.8 Hz), 7.13–7.24 (m, 6H), 7.32–7.33 (m, 2H).

**^13^C NMR** (151 MHz, CDCl_3_): δ 24.7 (CH_2_), 24.8 (CH_2_), 25.5 (CH_2_), 25.5 (CH_2_), 25.7 (CH_2_), 25.7 (CH_2_), 29.1 (CH_2_), 29.6 (CH_2_), 32.6 (CH_2_), 32.7 (CH_2_), 41.8 (CH), 48.4 (CH), 49.7 (CH_2_), 62.4 (CH), 126.0 (CH), 126.7 (CH), 128.1 (CH), 128.2 (CH), 128.4 (CH), 128.6 (CH), 129.5 (CH), 135.5 (C), 138.2 (C), 168.8 (C), 178.0 (C).

**IR** (KBr): cm^-1^ 3300, 3064, 3031, 2926, 2853, 1678, 1619, 1549, 1450, 700.

**HRMS** (ESI-TOF): *m/z* calcd for C_28_H_36_N_2_NaO_2_ ([M + Na]^+^): 455.2669; found: 455.2666.

**Synthesis of 1j**

According to the general procedure, citronellal (77.1 mg, 0.5 mmol), benzylamine (53.6 mg, 0.5 mmol), ursodeoxycholic acid (197 mg, 0.5 mmol), and cyclohexyl isocyanide (54.6 mg, 0.5 mmol) were converted into the corresponding Ugi adduct **1j** in 84 % yield (313 mg) a colorless amorphous solid after silica gel column chromatography (CHCl_3_/methanol = 99:1 to 49:1).

**1j**: 2-((*R*)-*N*-Benzyl-4-((3*R*,5*S*,7*S*,8*R*,9*S*,10*S*,13*R*,14*S*,17*R*)-3,7-dihydroxy-10,13-dimethylhexadecahydro-1*H*-cyclopenta[*a*]phenanthren-17-yl)pentanamido)-*N*-cyclohexyl-4,8-dimethylnon-7-enamide

**^1^H NMR** (400 MHz, CDCl_3_): δ 0.62 and 0.63 (s, 3H, diastereomers), 0.76–0.78 (m, 3H), 0.84 (t, 3H, *J* = 7.0 Hz), 0.93 (s, 3H), 0.93–2.01 (m, 41H), 1.56 and 1.57 (s, 3H, diastereomers), 1.65 and 1.67 (s, 3H, diastereomers), 2.10–2.32 (m, 2H), 3.54–3.71 (m, 3H), 4.55–4.66 (m, 2H), 4.98–5.08 (m, 2H), 6.34–6.40 (m, 1H), 7.16 (d, 2H, *J* = 7.6 Hz), 7.24–7.35 (m, 3H).

**^13^C NMR** (151 MHz, CDCl_3_): δ 12.1 and 12.1 (CH_3_, diastereomers), 17.5 and 17.6 (CH_3_, diastereomers), 18.3 and 18.4 (CH_3_, diastereomers), 19.2 and 19.6 (CH_3_, diastereomers), 21.1 (CH_2_), 23.4 (CH_3_), 24.7 (CH_2_), 24.7 (CH_2_), 25.2 and 25.2 (CH_2_, diastereomers), 25.5 (CH_2_), 25.6 and 25.7 (CH_3_, diastereomers), 26.8 (CH_2_), 28.5 (CH_2_), 29.4 and 29.5 (CH, diastereomers), 30.2 (CH_2_), 30.9 and 31.0 (CH_2_, diastereomers), 31.6 (CH_2_), 32.6 (CH_2_), 32.8 (CH_2_), 34.0 (C), 34.9 (CH_2_), 35.3 and 35.3 (CH, diastereomers), 35.5 (CH_2_), 36.8 (CH_2_), 37.1 (CH_2_), 37.3 (CH_2_), 39.2 (CH), 40.1 (CH_2_), 42.5 (CH), 43.5 (CH), 43.6 (C), 47.9 (CH), 48.1 (CH_2_), 55.0 (CH), 55.4 (CH), 55.8 (CH), 71.0 (CH), 71.0 (CH), 124.5 and 124.6 (CH, diastereomers), 125.8 and 125.8 (CH, diastereomers), 127.0 and 127.1 (CH, diastereomers), 128.5 (CH), 131.0 and 131.1 (C, diastereomers), 137.9 and 138.0 (C, diastereomers), 169.5 and 169.7 (C, diastereomers), 175.8 and 176.1 (C, diastereomers).

**IR** (KBr): cm^-1^ 3400, 3321, 2929, 2857, 1658, 1629, 1542, 1452, 1415, 1379, 1052, 731.

**HRMS** (ESI-TOF): *m/z* calcd for C_48_H_77_N_2_O_4_ ([M + H]^+^): 745.5878; found: 745.5876.

**Synthesis of 2**

**Extraction.** The pericarp of *Zanthoxylum piperitum* (5.03 g) was extracted with methanol (20 mL) in the dark at room temperature for 24 hours with vigorous stirring. The resulting solution was then filtered and concentrated *in vacuo* to afford the extract (1.32 g).

**Ugi-4CR.** To a solution of the methanol extract of *Z. piperitum* (1.32 g) in methanol (27.7 mL), benzylamine (981 mg, 9.16 mmol) was added. The mixture was stirred at room temperature for 1 hour. Then, acetic acid (550 mg, 9.16 mmol) was added and the solution was cooled to 0 °C. Then, TosMIC (1.79 g, 9.16 mmol) was added and stirred at 0 °C for 1 hour. After stirring at room temperature for 7 days, the reaction mixture was concentrated *in vacuo* to give the crude mixture (4.34 g).

**Isolation.** The crude mixture was chromatographed on a silica gel column eluted with a gradient of hexane/ethyl acetate (9:1, 2:1, 1:1, and 1:2) followed by a gradient of ethyl acetate/methanol (9:1 and 0:1) to give six fractions; Fr. 1 (53.2 mg), Fr. 2 (984 mg), Fr. 3 (341 mg), Fr. 4 (142 mg), Fr. 5 (556 mg), Fr. 6 (2.24 g). A part of Fr. 4 (132 mg) was rechromatographed on a silica gel column eluted with hexane/ethyl acetate (2:1 to 1:2) to provide Fr. 4-1 (1.90 mg), Fr. 4-2 (9.12 mg), Fr. 4-3 (1.73 mg), Fr. 4-4 (1.29 mg), Fr. 4-5 (0.11 mg), Fr. 4-6 (11.2 mg), Fr. 4-7 (5.86 mg), Fr. 4-8 (74.3 mg), Fr. 4-9 (4.36 mg), Fr. 4-10 (30.6 mg). Then, Fr. 4-6 was purified by preparative TLC eluted with CHCl_3_/methanol (49:1) to obtain **2** (5.28 mg) as a colorless amorphous solid.

**2**: 2-(*N*-Benzylacetamido)-4,8-dimethyl-*N*-(tosylmethyl)non-7-enamide

**^1^H NMR** (600 MHz, CDCl_3_): δ 0.74–0.76 (m, 3H), 0.97–1.19 (m, 3H), 1.46–1.88 (m, 4H), 1.54 and 1.56 (s, 3H, diastereomers), 1.65 and 1.67 (s, 3H, diastereomers), 2.13 and 2.14 (s, 3H, diastereomers), 2.44 and 2.44 (s, 3H, diastereomers), 4.37–4.46 (m, 2H), 4.54–4.67 (m, 2H), 4.92–4.96 (m, 1H), 4.98–5.00 (m, 1H), 7.12 (d, 2H, *J* = 8.4 Hz), 7.25–7.28 (m, 1H), 7.32–7.35 (m, 5H), 7.78 (d, 2H, *J* = 8.4 Hz).

**^13^C NMR** (151 Hz, CDCl_3_): δ 17.8 and 17.8 (CH_3_, diastereomers), 19.3 and 19.7 (CH_3_, diastereomers), 21.9 (CH_3_), 22.5 and 22.5 (CH_3_, diastereomers), 25.3 and 25.4 (CH_2_, diastereomers), 25.8 and 25.9 (CH_3_, diastereomers), 29.6 and 29.7 (CH, diastereomers), 34.9 and 35.2 (CH_2_, diastereomers), 36.9 and 37.5 (CH_2_, diastereomers), 49.5 and 49.5 (CH_2_, diastereomers), 55.6 and 56.0 (CH, diastereomers), 60.3 and 60.3 (CH_2_, diastereomers), 124.5 and 124.5 (CH, diastereomers), 126.1 and 126.2 (CH, diastereomers), 127.7 and 127.7 (CH, diastereomers), 129.0 (CH), 129.0 (CH), 130.0 and 130.0 (CH, diastereomers), 131.5 and 131.6 (C, diastereomers), 134.6 (C), 137.1 and 137.2 (C, diastereomers), 145.3 and 145.4 (C, diastereomers), 170.5 and 171.0 (C, diastereomers), 173.6 and 173.7 (C, diastereomers).

**IR** (KBr): cm^-1^ 3351, 3030, 2959, 2925, 2854, 1697, 1629, 1519, 1452, 1381, 1322, 1143.

**HRMS** (ESI-TOF): *m/z* calcd for C_28_H_38_N_2_NaO_4_S ([M + Na]^+^): 521.2445; found: 521.2445.

**Figure S10.** Key ^1^H–^1^H COSY and HMBC correlations of **2**.

**Synthesis of 4 and 5**

**From *Curcuma zedoaria* (CZ1)**

**Extraction.** The dried rhizome powder of *Curcuma zedoaria* (Uchida Wakanyaku, CZ1, 50 g) was extracted with methanol (200 mL) at room temperature for 24 hours with vigorous stirring. The resulting solution was filtered and concentrated *in vacuo* to afford the extract (1.85 g).

**Ugi reaction.** To a solution of the methanol extract of *C. zedoaria* (1.85 g) in methanol (3.90 mL, 0.5 M), benzylamine (209 mg, 1.95 mmol) was added. After stirring at room temperature for 1 hour, chloroacetic acid (184 mg, 1.95 mmol) was added. Then, the resulting solution was cooled to 0 °C and cyclohexyl isocyanide (213 mg, 1.95 mmol) was added at 0 °C and the reaction mixture was stirred at 0 °C for 1 hour and then at room temperature for 7 days. The resulting mixture was concentrated *in vacuo* to give the crude extract (2.35 g).

**Isolation.** The crude mixture was chromatographed on a silica gel column eluted with a gradient of hexane/ethyl acetate (9:1, 2:1, 1:1, and 1:2) followed by a gradient of ethyl acetate/methanol (9:1 and 0:1) to give six fractions; Fr. 1 (357 mg), Fr. 2 (515 mg), Fr. 3 (150 mg), Fr. 4 (140 mg), Fr. 5 (511 mg), Fr. 6 (615 mg). Fr. 3 (144 mg) was rechromatographed on a silica gel column eluted with CHCl_3_/ethyl acetate (47:3) to provide Fr. 3-1 (2.46 mg), Fr. 3-2 (58.4 mg), Fr. 3-3 (95.8 mg). Fr. 3-2 was further purified by preparative TLC eluted with CHCl_3_ and then with CHCl_3_/2-propanol (49:1) to afford **4** (37.5 mg) as a colorless amorphous solid. Fr. 5 (505 mg) was rechromatographed on a silica gel column eluted with CHCl_3_/ethyl acetate (9:1 to 7:3) to provide Fr. 5-1 (7.22 mg), Fr. 5-2 (39.3 mg), Fr. 5-3 (382 mg), and Fr. 5-4 (35.1 mg). Fr. 5-2 was further purified by preparative TLC eluted with dichloromethane/2-propanol (49:1) and then with CHCl_3_/acetone (49:1) to afford **5** (1.99 mg) as a colorless amorphous solid.

**From *Curcuma zedoaria* (CZ2)**

**Extraction.** The dried rhizome powder of *Curcuma zedoaria* (Keimeido, CZ2, 50 g) was extracted with methanol (200 mL) at room temperature for 24 hours with vigorous stirring. The resulting solution was filtered and concentrated *in vacuo* to afford the extract (2.27 g).

**Ugi reaction.** To a solution of the methanol extract of *C. zedoaria* (2.27 g) in methanol (4.40 mL, 0.5 M), benzylamine (236 mg, 2.20 mmol) was added. After stirring at room temperature for 1 hour, chloroacetic acid (208 mg, 2.20 mmol) was added. Then, the resulting solution was cooled to 0 °C and cyclohexyl isocyanide (240 mg, 2.20 mmol) was added at 0 °C and the reaction mixture was stirred at 0 °C for 1 hour and then at room temperature for 7 days. The resulting mixture was concentrated *in vacuo* to give the crude extract (2.90 g).

**Isolation.** The crude mixture was chromatographed on a silica gel column eluted with a gradient of hexane/ethyl acetate (9:1, 2:1, 1:1, and 1:2) followed by a gradient of ethyl acetate/methanol (9:1 and 0:1) to give six fractions; Fr. 1 (272 mg), Fr. 2 (426 mg), Fr. 3 (184 mg), Fr. 4 (171 mg), Fr. 5 (769 mg), Fr. 6 (1.05 g). Fr. 3 (175 mg) was rechromatographed on a silica gel column eluted with CHCl_3_/ethyl acetate (49:1) to afford Fr. 3-1 (7.03 mg), Fr. 3-2 (44.2 mg), and Fr. 3-3 (146 mg). Fr. 3-2 was further purified by preparative TLC eluted with CHCl_3_/ethyl acetate (24:1), with CHCl_3_/2-propanol (49:1), and then with 1,2-dichloroethane/ethanol (19:1) to afford **4** (4.03 mg) as a colorless amorphous solid.

**From *Curcuma longa* (CL1)**

**Extraction.** The dried rhizome powder of *Curcuma longa* (Keimeido, CL1, 50 g) was extracted with methanol (200 mL) at room temperature for 24 hours with vigorous stirring. The resulting solution was filtered and concentrated *in vacuo* to afford the extract (3.92 g).

**Ugi reaction.** To a solution of the methanol extract of *C. longa* (3.92 g) in methanol (11.1 mL, 0.5 M), benzylamine (594 mg, 5.54 mmol) was added. After stirring at room temperature for 1 hour, chloroacetic acid (524 mg, 5.54 mmol) was added. Then, the resulting solution was cooled to 0 °C and cyclohexyl isocyanide (605 mg, 2.20 mmol) was added at 0 °C and the reaction mixture was stirred at 0 °C for 1 hour and then at room temperature for 7 days. The resulting mixture was concentrated *in vacuo* to give the crude extract (5.56 g).

**Isolation.** The crude mixture was chromatographed on a silica gel column eluted with a gradient of hexane/ethyl acetate (9:1, 2:1, 1:1, and 1:2) followed by a gradient of ethyl acetate/methanol (9:1 and 0:1) to give six fractions; Fr. 1 (1.18 g), Fr. 2 (1.29 g), Fr. 3 (265 mg), Fr. 4 (323 mg), Fr. 5 (792 mg), Fr. 6 (1.71 g). Fr. 2 (1.28 g) was re-chromatographed on a silica gel column eluted with hexane/ethyl acetate (4:1, 7:3 to 3:2) to afford Fr. 3-1 (705 mg), Fr. 3-2 (80.3 mg), and Fr. 3-3 (333 mg). Fr. 3-2 was further purified by preparative TLC eluted with CHCl_3_/ethyl acetate (24:1) and then with CHCl_3_/methanol (99:1) to afford **4** (16.2 mg) as a colorless amorphous solid.

**4**: 2-(*N*-Benzyl-2-chloroacetamido)-*N*-cyclohexyl-2-methyl-4-((1*S*,6*R*,7*R*)-1-methyl-3-oxo-4-(propan-2-ylidene)bicyclo[4.1.0]heptan-7-yl)butanamide

**^1^H NMR** (400 MHz, CDCl_3_): δ 0.37–0.43 (m, 1H), 0.61–0.65 (m, 1H), 1.06 and 1.07 (s, 3H, diastereomers), 1.14–1.45 (m, 7H), 1.41 and 1.41 (s, 3H, diastereomers), 1.60–1.74 (m, 4H), 1.77 and 1.79 (s, 3H, diastereomers), 1.90–2.00 (m, 2H), 2.05–2.21 (m, 1H), 2.09 and 2.09 (s, 3H, diastereomers), 2.46–2.57 (m, 2H), 2.78–2.81 (m, 2H), 3.73–3.84 (m, 1H), 3.84–3.96 (m, 2H), 4.62 (d, 1H, *J* = 18.4 Hz), 4.76 (d, 1H, *J* = 18.4 Hz), 5.54–5.56 (m, 1H), 7.29 (t, 1H, *J* = 7.4 Hz), 7.40 (t, 2H, *J* = 7.4 Hz), 7.56 (d, 2H, *J* = 7.4 Hz).

**^13^C NMR** (150 MHz, CDCl_3_): δ 19.1 and 19.1 (CH_3_, diastereomers), 20.2 and 20.3 (C, diastereomers), 21.4 and 21.6 (CH_3_, diastereomers), 23.6 and 23.6 (CH_3_, diastereomers), 23.6 (CH_3_), 23.9 and 23.9 (CH_2_, diastereomers), 24.0 and 24.1 (CH, diastereomers), 24.3 (CH), 25.1 (CH_2_), 25.1 (CH_2_), 25.7 (CH_2_), 28.0 and 28.1 (CH_2_, diastereomers), 33.2 (CH_2_), 33.3 (CH_2_), 36.2 and 36.4 (CH_2_, diastereomers), 42.4 (CH_2_), 48.2 and 48.3 (CH_2_, diastereomers), 48.6 (CH), 49.0 (CH_2_), 65.6 and 65.6 (C), 126.1 (CH), 127.7 and 127.7 (CH, diastereomers), 128.1 and 128.2 (C, diastereomers), 129.2 and 129.2 (CH, diastereomers), 138.1 (C), 147.7 and 147.7 (C, diastereomers), 167.8 and 167.9 (C, diastereomers), 172.8 and 172.9 (C, diastereomers), 201.8 and 201.8 (C, diastereomers).

**IR** (KBr): cm^-1^ 3375, 2931, 2855, 1665, 1523, 1452, 1409, 1370, 753.

**HRMS** (ESI-TOF): *m/z* calcd for C_31_H_43_ClN_2_NaO_3_ ([M + Na]^+^): 549.2854; found 549.2856.

**Figure S11.** Key ^1^H ^1^H COSY and HMBC correlations of **4**.

**5**: 2-(*N*-Benzylacetamido)-*N*-cyclohexyl-2-methyl-4-((1*S*,6*R*,7*R*)-1-methyl-3-oxo-4-(propan-2-ylidene)bicyclo[4.1.0]heptan-7-yl)butanamide

**^1^H NMR** (600 MHz, CDCl_3_): δ 0.37–0.41 (m, 1H), 0.58–0.61 (m, 1H), 1.00 and 1.04 (s, 3H, diastereomers), 1.11–1.38 (m, 7H), 1.38 and 1.38 (s, 3H, diastereomers), 1.57–1.78 (m, 4H), 1.77 and 1.78 (s, 3H, diastereomers), 1.89–2.09 (m, 3H), 2.06 and 2.08 (s, 3H, diastereomers), 2.08 and 2.09 (s, 3H, diastereomers), 2.44–2.55 (m, 2H), 2.72–2.82 (m, 2H), 3.70–3.80 (m, 1H), 4.52–4.70 (m, 2H), 5.54–5.57 (m, 1H), 7.26–7.29 (m, 1H), 7.38 and 7.39 (t, 2H, *J* = 7.8 Hz, diastereomers), 7.50–7.52 (m, 2H).

**^13^C NMR** (150 MHz, CDCl_3_): δ 19.1 and 19.1 (CH_3_, diastereomers), 20.0 and 20.2 (C, diastereomers), 21.6 (CH_3_), 23.4 and 23.4 (CH_3_, diastereomers), 23.6 and 23.6 (CH_3_, diastereomers), 23.6 and 23.7 (CH_3_, diastereomers), 24.0 and 24.1 (CH, diastereomers), 24.1 and 24.2 (CH_2_, diastereomers), 24.5 and 24.6 (CH, diastereomers), 25.1 (CH_2_), 25.1 (CH_2_), 25.8 (CH_2_), 28.0 and 28.0 (CH_2_, diastereomers), 33.2 (CH_2_), 33.3 and 33.3 (CH_2_, diastereomers), 36.9 (CH_2_), 48.4 (CH), 49.0 (CH_2_), 49.1 (CH_2_), 65.2 and 65.2 (C, diastereomers), 126.4 (CH), 127.4 and 127.4 (CH, diastereomers), 128.2 and 128.2 (C, diastereomers), 129.1 and 129.1 (CH, diastereomers), 139.0 (C), 147.6 and 147.8 (C, diastereomers), 172.1 (C), 173.4 and 173.4 (C, diastereomers), 201.8 and 201.9 (C, diastereomers).

**IR** (film): cm^-1^ 3342, 2930, 2854, 1652, 1520, 1452, 1404, 1368, 753.

**HRMS** (ESI-TOF): *m/z* calcd for C_31_H_44_N_2_NaO_3_ ([M + Na]^+^): 515.3244; found: 515.3245.

**Isolation of curcumenone (6)**

**From *Curcuma zedoaria* (CZ1)**

**Extraction.** The dried rhizome powder of *Curcuma zedoaria* (Uchida Wakanyaku, CZ1, 50 g) was extracted with methanol (200 mL) at room temperature for 24 hours with vigorous stirring. The resulting solution was filtered and concentrated *in vacuo* to afford the extract (1.56 g).

**Isolation.** The extract was chromatographed on a silica gel column eluted with a gradient of hexane/ethyl acetate (9:1, 2:1, 1:1, and 1:2) followed by a gradient of ethyl acetate/methanol (9:1 and 0:1) to give six fractions; Fr. 1 (282 mg), Fr. 2 (441 mg), Fr. 3 (75.3 mg), Fr. 4 (95.9 mg), Fr. 5 (246 mg), and Fr. 6 (303 mg). Fr. 2 was re-chromatographed on a silica gel column eluted with hexane/ethyl acetate (4:1) to afford Fr. 2-1 (8.68 mg), Fr. 2-2 (293 mg), Fr. 2-3 (15.5 mg), Fr. 2-4 (86.2 mg), and Fr. 2-5 (208 mg). Then, Fr. 2-4 was purified by preparative TLC eluted with CHCl_3_/hexane (47:3) to provide Fr. 2-4-1 (59.8 mg). Fr. 2-4-1 was further purified by preparative TLC eluted with 1,2-dichloroethane/2-propanol (23:2) and then with 1,2-dichloroethane/diethyl ether (47:3) to afford curcumenone (**6**)^2^ (30.2 mg) as a colorless oil.

**From *Curcuma zedoaria* (CZ2)**

**Extraction.** The dried rhizome powder of *Curcuma zedoaria* (Keimeido, CZ2, 50 g) was extracted with methanol (200 mL) at room temperature for 24 hours with vigorous stirring. The resulting solution was filtered and concentrated *in vacuo* to afford the extract (2.59 g).

**Isolation.** The extract was chromatographed on a silica gel column eluted with a gradient of hexane/ethyl acetate (9:1, 2:1, 1:1, and 1:2) followed by a gradient of ethyl acetate/methanol (9:1 and 0:1) to give six fractions; Fr. 1 (446 mg), Fr. 2 (460 mg), Fr. 3 (141 mg), Fr. 4 (127 mg), Fr. 5 (446 mg), and Fr. 6 (873 mg). Fr. 2 (449 mg) was rechromatographed on a silica gel column eluted with hexane/ethyl acetate (100:0 to 95:5) to provide Fr. 2-1 (13.9 mg), Fr. 2-2 (66.1 mg), Fr. 2-3 (83.2 mg), Fr. 2-4 (101 mg), Fr. 2-5 (22.9 mg), and Fr. 2-6 (150 mg). Then, Fr. 2-5 was purified by preparative TLC eluted with CHCl_3_ to provide Fr. 2-5-1 (14.2 mg). Fr. 2-5-1 was further purified by preparative TLC eluted with 1,2-dichloroethane/ethyl acetate (9:1) to afford curcumenone (**6**) (6.33 mg) as a colorless oil.

**From *Curcuma longa* (CL1)**

**Extraction.** The dried rhizome powder of *Curcuma longa* (Keimeido, CL1, 50 g) was extracted with methanol (200 mL) at room temperature for 24 hours with vigorous stirring. The resulting solution was filtered and concentrated *in vacuo* to afford the extract (3.74 g).

**Isolation.** The extract was chromatographed on a silica gel column eluted with a gradient of hexane/ethyl acetate (9:1, 2:1, 1:1, and 1:2) followed by a gradient of ethyl acetate/methanol (9:1 and 0:1) to give six fractions; Fr. 1 (1.19 g), Fr. 2 (611 mg), Fr. 3 (272 mg), Fr. 4 (237 mg), Fr. 5 (551 mg), and Fr. 6 (672 mg). Fr. 2 (607 mg) was rechromatographed on a silica gel column eluted with hexane/ethyl acetate (100:0 to 79:21) to give Fr. 2-1 (4.55 mg), Fr. 2-2 (232 mg), Fr. 2-3 (98.4 mg), and Fr. 2-4 (254 mg). Then, Fr. 2-3 was again chromatographed on a silica gel column eluted with CHCl_3_/methanol (100:0 to 94:6) to afford Fr. 2-3-1 (2.35 mg), Fr. 2-3-2 (3.38 mg), Fr. 2-3-3 (22.0 mg), and Fr. 2-3-4 (66.6 mg). Fr. 2-3-3 and Fr. 2-3-4 were further purified by preparative TLC eluted with CHCl_3_ and then with 1,2-dichloroethane/ethyl acetate (9:1) to obtain curcumenone (**6**) (14.8 mg) as a colorless oil.

**Synthesis of 7(a–c)**

**Extraction.** The dried rhizome powder of *Curcuma zedoaria* (Uchida Wakanyaku, CZ1, 50 g) was extracted with methanol (200 mL) at room temperature for 24 hours with vigorous stirring. The resulting solution was filtered and concentrated *in vacuo* to afford the extract (1.70 g).

**Ugi reaction.** To a solution of the methanol extract of *C. zedoaria* (1.70 g) in methanol (3.58 mL, 0.5 M), 1-aminopyrene (389 mg, 1.79 mmol) was added. After stirring at room temperature for 1 hour, chloroacetic acid (169 mg, 1.79 mmol) was added. Then, the resulting solution was cooled to 0 °C and cyclohexyl isocyanide (195 mg, 1.79 mmol) was added at 0 °C and the reaction mixture was stirred at 0 °C for 1 hour and then at room temperature for 7 days. The resulting mixture was concentrated *in vacuo* to give the crude extract (2.45 g).

**Isolation.** The crude mixture was chromatographed on a silica gel column eluted with a gradient of CHCl_3_/ethyl acetate (19:1, 4:1, 1:1, and 1:2) followed by a gradient of CHCl_3_/methanol (4:1 and 0:1) to give six fractions; Fr. 1 (1.01 g), Fr. 2 (357 mg), Fr. 3 (195 mg), Fr. 4 (125 mg), Fr. 5 (524 mg), Fr. 6 (235 mg). Fr. 1 (995 mg) was rechromatographed on a silica gel column eluted with hexane/ethyl acetate (7:3) to obtain Fr. 1-1 (653 mg), Fr. 1-2 (103 mg), Fr. 1-3 (71.6 mg), Fr. 1-4 (96.7 mg), and Fr. 1-5 (141 mg). Fr. 1-4 was then purified by preparative TLC eluted with dichloromethane/ethyl acetate (9:1) to provide Fr. 1-4-1 (26.2 mg), Fr. 1-4-2 (11.1 mg), and Fr. 1-4-3 (9.27 mg). Fr. 1-4-1 was further purified by preparative TLC eluted with CHCl_3_/acetone (49:1) to afford a 1:1 diastereomeric mixture of **7a** (25.1 mg) a colorless amorphous solid. Fr. 1-4-2 was further purified by preparative TLC eluted with CHCl_3_/2-propanol (49:1) and then with CHCl_3_/acetone (49:1) to obtain **7b** (5.06 mg) a colorless amorphous solid. Fr. 1-4-3 was further purified by preparative TLC eluted with CHCl_3_/acetone (49:1) and then with CHCl_3_/diethyl ether (47:3) to afford **7c** (4.40 mg) as a colorless amorphous solid.

**7**: 2-(2-Chloro-*N*-(pyren-1-yl)acetamido)-*N*-cyclohexyl-2-methyl-4-((1*S*,6*R*,7*R*)-1-methyl-3-oxo-4-(propan-2-ylidene)bicyclo[4.1.0]heptan-7-yl)butanamide

**7a**; **^1^H NMR** (600 MHz, CDCl_3_): δ -0.11–-0.08 and -0.04–-0.01 (m, 1H, diastereomers), 0.13–0.15 and 0.30–0.31 (m, 1H, diastereomers), 0.79 and 0.86 (s, 3H, diastereomers), 0.99–1.36 (m, 6H), 1.17 and 1.36 (s, 3H, diastereomers), 1.39–1.49 (m, 2H), 1.65–1.67 (m, 1H), 1.74-1.83 (m, 3H), 1.77 and 1.80 (s, 3H, diastereomers), 1.78 and 1.83 (s, 3H, diastereomers), 1.95–2.01 (m, 1H), 2.04–2.36 (m, 4H), 2.41 (d, 1H, *J* = 17.4 Hz), 3.44 (d, 0.5H, *J* = 13.8 Hz), 3.45 (d, 0.5H, *J* = 14.4 Hz), 3.59 (d, 0.5H, *J* = 14.4 Hz), 3.60 (d, 0.5H, *J* = 13.8 Hz), 3.92–4.00 (m, 1H), 5.87 and 5.91 (br d, 1H, *J* = 7.8 Hz, diastereomers), 7.84–7.86 (m, 1H), 8.07–8.13 (m, 2H), 8.19–8.21 (m, 2H), 8.27–8.30 (m, 3H), 9.19 and 9.22 (d, 1H, *J* = 9.0 Hz, diastereomers).

**^13^C NMR** (151 MHz, CDCl_3_): δ 19.0 and 19.0 (CH_3_, diastereomers), 19.7 and 20.0 (C, diastereomers), 19.9 and 19.9 (CH_3_, diastereomers), 22.8 and 23.0 (CH_3_, diastereomers), 23.3 and 23.3 (CH_3_, diastereomers), 24.0 and 24.4 (CH_2_, diastereomers), 24.0 and 24.1 (CH, diastereomers), 24.2 and 24.2 (CH, diastereomers), 25.1 (CH_2_), 25.1 (CH_2_), 25.8 and 25.8 (CH_2_, diastereomers), 27.5 (CH_2_), 33.1 (CH_2_), 33.5 and 33.7 (CH_2_, diastereomers), 38.1 and 38.3 (CH_2_, diastereomers), 43.6 (CH_2_), 48.6 and 48.6 (CH_2_, diastereomers), 49.0 (CH), 67.2 and 67.2 (C, diastereomers), 123.2 and 123.4 (CH, diastereomers), 124.4 (C), 125.0 and 125.0 (CH, diastereomers), 125.6 (C), 126.3 and 126.3 (CH, diastereomers), 126.3 and 126.4 (CH, diastereomers), 126.9 and 126.9 (CH, diastereomers), 127.0 and 127.0 (CH, diastereomers), 127.2 and 127.3 (CH, diastereomers), 128.0 (C), 129.1 and 129.1 (CH, diastereomers), 130.0 and 130.1 (CH, diastereomers), 131.1 and 131.1 (C, diastereomers), 131.2 (C), 131.4 and 131.4 (C, diastereomers), 131.5 and 131.6 (C, diastereomers), 132.2 and 132.3 (C, diastereomers), 147.1 and 147.1 (C, diastereomers), 166.9 and 167.0 (C, diastereomers), 172.4 and 172.5 (C, diastereomers), 201.6 and 201.7 (C, diastereomers).

**IR** (KBr): cm^-1^ 3398, 2929, 2854, 1673, 1523, 1453, 1437, 1366, 851, 753.

**HRMS** (ESI-TOF): *m/z* calcd for C_40_H_45_ClN_2_NaO_3_ ([M + Na]^+^): 659.3011; found: 659.3013.

**7b**; **^1^H NMR** (600 MHz, CDCl_3_): δ 0.07–0.11 (m, 1H), 0.43–0.45 (m, 1H), 0.89 (s, 3H), 1.17–1.35 (m, 6H), 1.41–1.47 (m, 2H), 1.56 (s, 3H), 1.64–1.68 (m, 1H), 1.72 (s, 3H), 1.74–1.84 (m, 3H), 1.92 (s, 3H), 2.04–2.10 (m, 2H), 2.26 (d, 1H, *J* = 15.9 Hz), 2.36 (d, 1H, *J* =15.9 Hz), 2.54 (d, 1H, *J* = 16.2 Hz), 2.62 (d, 1H, *J* = 16.2 Hz), 3.52 (d, 1H, *J* = 13.5 Hz), 3.60 (d, 1H, *J* = 13.5 Hz), 3.89–3.95 (m, 1H), 6.00 (br d, 1H, *J* = 7.8 Hz), 8.09 (t, 1H, *J* = 7.8 Hz), 8.12 (d, 1H, *J* = 9.0 Hz), 8.19 (d, 1H, *J* = 9.0 Hz), 8.22–8.24 (m, 2H), 8.26–8.33 (m, 3H), 8.39 (d, 1H, *J* = 9.0 Hz).

**^13^C NMR** (151 MHz, CDCl_3_): δ 19.2 (CH_3_), 20.1 (C), 21.7 (CH_3_), 23.3 (CH_3_), 23.4 (CH_3_), 24.1 (CH), 24.3 (CH_2_), 24.5 (CH), 25.1 (CH_2_), 25.1 (CH_2_), 25.8 (CH_2_), 27.9 (CH_2_), 33.2 (CH_2_), 33.5 (CH_2_), 38.8 (CH_2_), 44.0 (CH_2_), 48.8 (CH_2_), 49.1 (CH), 67.3 (C), 122.0 (CH), 124.6 (C), 125.1 (CH), 125.5 (C), 126.2 (CH), 126.5 (CH), 126.9 (CH), 127.2 (CH), 128.1 (C), 129.0 (CH), 129.2 (CH), 130.1 (CH), 130.5 (C), 130.9 (C), 131.3 (C), 132.1 (C), 147.3 (C), 167.4 (C), 172.6 (C), 201.7 (C).

**IR** (KBr): cm^-1^ 3444, 2926, 2852, 1671, 1516, 1454, 1435, 1369, 853, 754.

**HRMS** (ESI-TOF): *m/z* calcd for C_40_H_45_ClN_2_NaO_3_ ([M + Na]^+^): 659.3011; found: 659.3011.

**7c**; **^1^H NMR** (600 MHz, CDCl_3_): δ 0.06–0.12 (m, 2H), 0.79 (s, 3H), 1.09–1.15 (m, 1H), 1.21–1.36 (m, 4H), 1.41–1.48 (m, 3H), 1.58 (s, 3H), 1.65–1.68 (m, 1H), 1.72 (s, 3H), 1.75–1.80 (m, 3H), 1.96 (s, 3H), 2.05–2.10 (m, 2H), 2.28 (d, 1H, *J* = 16.2 Hz), 2.33 (d, 1H, *J* = 16.2 Hz), 2.47 (d, 1H, *J* = 16.2 Hz), 2.53 (d, 1H, *J* = 16.2 Hz), 3.52 (d, 1H, *J* = 13.8 Hz), 3.58 (d, 1H, *J* = 13.8 Hz), 3.87–3.94 (m, 1H), 6.05 (br d, 1H, *J* = 7.2 Hz), 8.09 (t, 1H, *J* = 7.2 Hz), 8.13 (d, 1H, *J* = 9.0 Hz), 8.19 (d, 1H, *J* = 9.0 Hz), 8.23 (d, 1H, J = 9.0 Hz), 8.24 (d, 1H, J = 9.0 Hz), 8.27 (d, 1H, *J* = 7.2 Hz), 8.29 (d, 1H, *J* = 7.2 Hz), 8.35 (d, 1H, *J* = 9.0 Hz), 8.37 (d, 1H, *J* = 9.0 Hz).

**^13^C NMR** (151 MHz, CDCl_3_): δ 19.0 (CH_3_), 19.9 (C), 21.4 (CH_3_), 23.4 (CH_3_), 23.5 (CH_3_), 24.0 (CH), 24.2 (CH), 24.3 (CH_2_), 25.1 (CH_2_), 25.1 (CH_2_), 25.8 (CH_2_), 27.7 (CH_2_), 33.2 (CH_2_), 33.4 (CH_2_), 38.6 (CH_2_), 44.0 (CH_2_), 48.8 (CH_2_), 49.1 (CH), 67.5 (C), 121.9 (CH), 124.6 (C), 125.1 (CH), 125.5 (C), 126.2 (CH), 126.5 (CH), 126.9 (CH), 127.2 (CH), 128.0 (C), 129.0 (CH), 129.3 (CH), 130.1 (CH), 130.4 (C), 130.8 (C), 131.3 (C), 132.0 (C), 132.2 (C), 147.4 (C), 167.5 (C), 172.9 (C), 201.6 (C).

**IR** (KBr): cm^-1^ 3356, 2928, 2853, 1676, 1517, 1453, 1436, 1367, 853, 755.

**HRMS** (ESI-TOF): *m/z* calcd for C_40_H_45_ClN_2_NaO_3_ ([M + Na]^+^): 659.3011; found: 659.3012.

**Figure S12.** Key ^1^H ^1^H COSY and HMBC correlations of **7**.

**Synthesis of 8a–8d and 9**

**Ugi reaction.** To a solution of citronellal (77.1 mg, 0.5 mmol) in methanol (1.0 mL, 0.5 M), benzylamine (53.7 mg, 0.5 mmol) was added. The mixture was stirred at room temperature for 1 hour. Then, castor oil fatty acids (CO-FA S) (155 mg) was added and the resulting mixture was cooled to 0 °C. Then, cyclohexyl isocyanide (54.6 mg, 0.5 mmol) was added at 0 °C and stirred at 0 °C for 1 hour and then at room temperature for 7 days. The reaction mixture was then concentrated *in vacuo* and the residue was dissolved with CHCl_3_ and washed with 1 M HCl, saturated NaHCO_3_ aq., and water. The CHCl_3_ layer was concentrated *in vacuo* to give the crude mixture (283 mg).

**Isolation.** The crude mixture was chromatographed on a silica gel column eluted with hexane/ethyl acetate (6:1) to give four fractions; Fr. 1 (22.1 mg), Fr. 2 (7.06 mg), Fr. 3 (12.7 mg), Fr. 4 (54.7 mg), and **8a** (143 mg) as a colorless oil. By ^1^H NMR analysis, Fr. 1 was found to be a 1:1 mixture of **8c** and **8d**. Fr. 2 was further purified by preparative TLC eluted with CHCl_3_/acetone (97:3) to provide Fr. 2-1 (4.08 mg). Fr. 3 was further purified by preparative TLC eluted with CHCl_3_/acetone (49:1) to provide Fr. 3-1 (3.09 mg) and Fr. 3-2 (5.29 mg). A combined mixture of Fr. 2-1 and Fr. 3-1 was then purified by preparative TLC eluted with 1,2-dichloroethane/*n*-butanol (19:1) to obtain **8b** (5.59 mg) as a colorless oil. **8a**–**8d** were obtained as a 1:1 mixture of diastereomers. Fr. 4 was purified by preparative TLC eluted with CHCl_3_ to afford **9** (4.20 mg) a colorless amorphous solid.

**8a**: (12*R*,*Z*)-*N*-Benzyl-*N*-(1-(cyclohexylamino)-4,8-dimethyl-1-oxonon-7-en-2-yl)-12-hydroxyoctadec-9-enamide

**^1^H NMR** (600 MHz, CDCl_3_): δ 0.83 and 0.85 (d, 3H, *J* = 6.6 Hz, diastereomers), 0.88 (t, 3H, *J* = 6.6 Hz), 1.04–1.37 (m, 24.5H), 1.43-1.48 (m, 3H), 1.55–1.70 (m, 5H), 1.55 and 1.57 (s, 3H, diastereomers), 1.65 and 1.67 (s, 3H, diastereomers), 1.77–2.05 (6.5H), 2.19–2.28 (m, 4H), 3.59–3.63 (m, 1H), 3.65-3.69 (m, 1H), 4.57-4.65 (m, 2H), 4.98-5.04 (m, 2H), 5.37–5.42 (m, 1H), 5.52–5.56 (m, 1H), 6.38–6.43 (m, 1H), 7.15 (d, 2H, *J* = 7.8 Hz), 7.23–7.26 (m, 1H), 7.32 and 7.32 (t, 2H, *J* = 7.8 Hz, diastereomers).

**^13^C NMR** (151 MHz, CDCl_3_): δ 14.2 (CH_3_), 17.7 and 17.8 (CH_3_, diastereomers), 19.4 and 19.8 (CH_3_, diastereomers), 22.7 (CH_2_), 24.8 (CH_2_), 24.8 (CH_2_), 25.4 and 25.4 (CH_2_, diastereomers), 25.5 and 25.6 (CH_2_, diastereomers), 25.6 (CH_2_), 25.8 and 25.8 (CH_3_, diastereomers), 25.8 (CH_2_), 27.5 (CH_2_), 29.2 (CH_2_), 29.3 (CH_2_), 29.3 (CH_2_), 29.5 (CH_2_), 29.7 (CH), 29.7 (CH_2_), 31.9 (CH_2_), 32.9 (CH_2_), 33.0 (CH_2_), 34.0 and 34.1 (CH_2_, diastereomers), 35.4 and 35.8 (CH_2_, diastereomers), 35.5 (CH_2_), 37.0 and 37.0 (CH_2_, diastereomers), 37.1 and 37.3 (CH_2_, diastereomers), 48.0 (CH), 48.3 and 48.5 (CH_2_, diastereomers), 56.0 and 56.0 (CH, diastereomers), 71.6 and 71.6 (CH, diastereomers), 124.7 and 124.7 (CH, diastereomers), 125.4 and 125.4 (CH, diastereomers), 126.0 and 126.1 (CH, diastereomers), 127.3 and 127.3 (CH, diastereomers), 128.7 (CH), 131.2 and 131.3 (C, diastereomers), 133.3 (CH), 138.0 and 138.1 (C, diastereomers), 169.9 and 170.1 (C, diastereomers), 175.6 and 175.8 (C, diastereomers).

**IR** (film): cm^-1^ 3429, 3315, 2927, 2854, 1658, 1630, 1542, 1452, 1416, 1378, 1078, 755, 727, 697.

**HRMS** (ESI-TOF): *m/z* calcd for C_42_H_70_N_2_NaO_3_ ([M + Na]^+^): 673.5279; found: 673.5277.

**Figure S13.** Key ^1^H ^1^H COSY and HMBC correlations of **8a**.

**8b**: (7*R*,*Z*)-18-(Benzyl(1-(cyclohexylamino)-4,8-dimethyl-1-oxonon-7-en-2-yl)amino)-18-oxooctadec-9-en-7-yl (12*R*,*Z*)-12-hydroxyoctadec-9-enoate

**^1^H NMR** (600 MHz, CDCl_3_): δ 0.83 and 0.85 (d, 3H, *J* = 6.6 Hz, diastereomers), 0.87 (t, 3H, *J* = 7.2Hz), 0.88 (t, 3H, *J* = 7.2 Hz), 1.05–1.37 (m, 40H), 1.41–1.70 (m, 14H), 1.55 and 1.57 (s, 3H, diastereomers), 1.65 and 1.67 (s, 3H, diastereomers), 1.77–1.93 (m, 3H), 1.97–2.00 (m, 2H), 2.03–2.06 (m, 2H), 2.20–2.31 (m, 8H), 3.59–3.70 (m, 2H), 4.55–4.65 (m, 2H), 4.87 (quintet, 1H, *J* = 6.6 Hz), 4.98–5.05 (m, 2H), 5.29–5.33 (m, 1H), 5.38–5.46 (m, 2H), 5.53–5.57 (m, 1H), 6.40 and 6.44 (br d, 1H, *J* = 8.1 Hz, diastereomers), 7.15 (d, 2H, *J* = 7.8 Hz), 7.25 (t, 1H, *J* = 7.8 Hz), 7.31 and 7.32 (t, 2H, *J* = 7.8 Hz).

**^13^C NMR** (151 MHz, CDCl_3_): δ 14.2 (CH_3_), 14.2 (CH_3_), 17.8 and 17.8 (CH_3_, diastereomers), 19.4 and 19.8 (CH_3_, diastereomers), 22.7 (CH_2_), 22.8 (CH_2_), 24.8 (CH_2_), 24.8 (CH_2_), 25.2 (CH_2_), 25.5 (CH_2_), 25.5 (CH_2_), 25.6 (CH_2_), 25.7 (CH_2_), 25.8 (CH_3_), 25.9 (CH_2_), 27.5 (CH_2_), 27.5 (CH_2_), 29.3 (CH_2_), 29.3 (CH_2_), 29.3 (CH_2_), 29.3 (CH_2_), 29.3 (CH_2_), 29.4 (CH_2_), 29.5 (CH_2_), 29.5 (CH_2_), 29.7 (CH_2_), 29.7 (CH_2_), 29.8 (CH), 31.9 (CH_2_), 32.0 (CH_2_), 32.1 (CH_2_), 32.9 (CH_2_), 33.1 (CH_2_), 33.8 (CH_2_), 34.1 and 34.2 (CH_2_, diastereomers), 34.8 (CH_2_), 35.4 and 35.8 (CH_2_, diastereomers), 35.5 (CH_2_), 37.0 (CH_2_), 37.1 and 37.4 (CH_2_, diastereomers), 48.1 (CH), 48.4 and 48.5 (CH_2_, diastereomers), 56.0 and 56.1 (CH, diastereomers), 71.6 (CH), 73.8 (CH), 124.5 (CH), 124.7 and 124.8 (CH, diastereomers), 125.4 (CH), 126.1 and 126.2 (CH, diastereomers), 127.3 and 126.4 (CH, diastereomers), 128.8 (CH), 131.3 and 131.4 (C, diastereomers), 132.6 (CH), 133.5 (CH), 138.0 and 138.1 (C, diastereomers), 169.9 and 170.2 (C, diastereomers), 173.7 (C), 175.7 and 175.9 (C, diastereomers).

**IR** (film): cm^-1^ 3444, 3317, 3008, 2927, 2854, 1733, 1671, 1629, 1543, 1452, 1415, 1378, 1179, 1079, 727, 697.

**HRMS** (ESI-TOF): *m/z* calcd for C_60_H_102_N_2_NaO_5_ ([M + Na]^+^): 953.7681; found: 953.7682.

**Figure S14.** Key ^1^H ^1^H COSY and HMBC correlations of **8b**.

**8c**: *N*-Benzyl-*N*-(1-(cyclohexylamino)-4,8-dimethyl-1-oxonon-7-en-2-yl)oleamide

**^1^H** **NMR** (600 MHz, CDCl_3_,): δ 0.82 and 0.85 (d, 3H, *J* = 6.6 Hz, diastereomers), 0.88 (t, 3H, *J* = 7.2 Hz), 1.04–1.37 (m, 28H), 1.55 and 1.57 (s, 3H, diastereomers), 1.55–1.70 (m, 8H), 1.65 and 1.67 (s, 3H, diastereomers), 1.78–2.01 (m, 7H), 2.20–2.28 (m, 2H), 3.63–3.70 (m, 1H), 4.55–4.64 (m, 2H), 4.98–5.03 (m, 2H), 5.30–5.36 (m, 2H), 6.36 and 6.39 (br d, 1H, *J* = 7.5 Hz, diastereomers), 7.16 (d, 2H, *J* = 7.2 Hz), 7.25 (t, 1H, *J* = 7.2 Hz), 7.32 and 7.32 (t, 2H, *J* = 7.2 Hz, diastereomers).

**^13^C** **NMR** (151 MHz, CDCl_3_): δ 14.2 (CH_3_), 17.7 and 17.8 (CH_3_, diastereomers), 19.4 and 19.7 (CH_3_, diastereomers), 22.8 (CH_2_), 24.8 (CH_2_), 24.8 (CH_2_), 25.4 and 25.4 (CH_2_, diastereomers), 25.6 and 25.6 (CH_2_, diastereomers), 25.6 (CH_2_), 25.8 and 25.8 (CH_3_, diastereomers), 27.3 (CH_2_), 27.3 (CH_2_), 29.2 (CH_2_), 29.4 (CH_2_), 29.4 (CH_2_), 29.4 (CH_2_), 29.4 (CH_2_), 29.4 (CH_2_), 29.6 (CH_2_), 29.7 (CH), 29.8 (CH_2_), 29.9 (CH_2_), 32.0 (CH_2_), 32.9 (CH_2_), 33.0 (CH_2_), 34.1 and 34.1 (CH_2_, diastereomers), 35.3 and 35.7 (CH_2_, diastereomers), 37.1 and 37.3 (CH_2_, diastereomers), 48.0 (CH), 48.3 and 48.5 (CH_2_, diastereomers), 56.0 and 56.1 (CH, diastereomers), 124.7 and 124.7 (CH, diastereomers), 126.1 and 126.1 (CH, diastereomers), 127.3 and 127.3 (CH, diastereomers), 128.8 (CH), 129.8 (CH), 130.1 (CH), 131.3 and 131.4 (C, diastereomers), 138.0 and 138.1 (C, diastereomers), 169.9 and 170.1 (C, diastereomers), 175.7 and 175.9 (C, diastereomers).

**IR** (film): cm^-1^ 3317, 2926, 2854, 1674, 1630, 1536, 1452, 1416, 1378, 755, 727, 696.

**HRMS** (ESI-TOF): *m/z* calcd for C_42_H_71_N_2_O_2_ ([M + H]^+^): 635.5510; found: 635.5510.

**Figure S15.** Key ^1^H ^1^H COSY and HMBC correlations of **8c**.

**8d**: (9*Z*,12*Z*)-*N*-Benzyl-*N*-(1-(cyclohexylamino)-4,8-dimethyl-1-oxonon-7-en-2-yl)octadeca-9,12-dienamide

**^1^H NMR** (600 MHz, CDCl_3_): δ 0.82 and 0.85 (d, 3H, *J* = 6.6 Hz, diastereomers), 0.89 (t, 3H, *J* = 7.2 Hz), 1.04–1.38 (m, 22H), 1.53–1.71 (m, 8H), 1.55 and 1.56 (s, 3H, diastereomers), 1.65 and 1.67 (s, 3H, diastereomers), 1.77–2.06 (m, 7H), 2.20–2.28 (m, 2H), 2.75–2.77 (m, 2H), 3.63–3.70 (m, 1H), 4.57–4.64 (m, 2H), 4.98–5.03 (m, 2H), 5.30–5.40 (m, 4H), 6.36 and 6.39 (br d, 1H, *J* = 7.8 Hz), 7.16 (d, 2H, *J* = 7.2 Hz), 7.24–7.26 (m, 1H), 7.32 and 7.32 (t, 2H, *J* = 7.2 Hz).

**^13^C NMR** (151 MHz, CDCl_3_): δ 14.2 (CH_3_), 17.7 and 17.8 (CH_3_, diastereomers), 19.4 and 19.7 (CH_3_, diastereomers), 22.7 (CH_2_), 24.8 (CH_2_), 24.8 (CH_2_), 25.4 and 25.4 (CH_2_, diastereomers), 25.6 and 25.6 (CH_2_, diastereomers), 25.6 (CH_2_), 25.7 (CH_2_), 25.8 and 25.8 (CH_3_, diastereomers), 27.3 (CH_2_), 27.3 (CH_2_), 29.2 (CH_2_), 29.4 (CH_2_), 29.4 (CH_2_), 29.4 (CH_2_), 29.7 (CH_2_), 29.7 (CH), 31.6 (CH_2_), 32.9 (CH_2_), 33.0 (CH_2_), 34.1 and 34.1 (CH_2_, diastereomers), 35.4 and 35.7 (CH_2_, diastereomers), 37.1 and 37.3 (CH_2_, diastereomers), 48.0 (CH), 48.3 and 48.5 (CH_2_, diastereomers), 56.0 and 56.1 (CH, diastereomers), 124.7 and 124.7 (CH, diastereomers), 126.1 and 126.1 (CH, diastereomers), 127.3 and 127.3 (CH, diastereomers), 128.0 (CH), 128.1 (CH), 128.8 (CH), 130.1 (CH), 130.3 (CH), 131.3 and 131.4 (C, diastereomers), 138.0 and 138.1 (C, diastereomers), 169.9 and 170.1 (C, diastereomers), 175.7 and 175.8 (C, diastereomers).

**IR** (film): cm^-1^ 3317, 3008, 2927, 2854, 1675, 1631, 1537, 1452, 1417, 1378, 727, 697.

**HRMS** (ESI-TOF): *m/z* calcd for C_42_H_68_N_2_NaO_2_ ([M + Na]^+^): 655.5173; found: 655.5171.

**Figure S16.** Key ^1^H ^1^H COSY and HMBC correlations of **8d**.

**9**: (12*R*,*Z*)-*N*-Benzyl-*N*-(2-(cyclohexylamino)-2-oxo-1-phenylethyl)-12-hydroxyoctadec-9-enamide

**^1^H NMR** (600 MHz, CDCl_3_): δ 0.88 (t, 3H, *J* = 6.9 Hz), 1.03–1.69 (m, 28H), 1.87–1.92 (m, 2H), 2.00–2.05 (m, 2H), 2.19–2.24 (m, 3H), 2.31–2.36 (m, 1H), 3.59–3.63 (m, 1H), 3.77–3.82 (m, 1H), 4.54 (d. 1H, *J* = 17.4 Hz), 4.73 (d, 1H, *J* = 17.4 Hz), 5.37–5.42 (m, 1H), 5.52–5.56 (m, 1H), 5.64 (br s, 1H), 5.93 (s, 1H), 6.99 (d, 2H, *J* = 7.2 Hz), 7.12–7.18 (m, 3H), 7.23–7.25 (m, 3H), 7.34–7.35 (m, 2H).

**^13^C NMR** (151 MHz, CDCl_3_): δ 14.1 (CH_3_), 22.7 (CH_2_), 24.8 (CH_2_), 24.8 (CH_2_), 25.2 (CH_2_), 25.5 (CH_2_), 25.8 (CH_2_), 27.4 (CH_2_), 29.1 (CH_2_), 29.2 (CH_2_), 29.4 (CH_2_), 29.6 (CH_2_), 31.9 (CH_2_), 32.8 (CH_2_), 32.8 (CH_2_), 34.0 (CH_2_), 35.4 (CH_2_), 36.9 (CH_2_), 48.6 (CH), 50.0 (CH_2_), 62.6 (CH), 71.5 (CH), 125.3 (CH), 126.1 (CH), 126.8 (CH), 128.3 (CH), 128.4 (CH), 128.6 (CH), 129.7 (CH), 133.2 (CH), 135.4 (C), 137.9 (C), 168.8 (C), 175.2 (C).

**IR** (film): cm^-1^ 3421, 3308, 3063, 3030, 3006, 2928, 2854, 1630, 1544, 1453, 1408, 728, 698.

**HRMS** (ESI-TOF): *m/z* calcd for C_39_H_58_N_2_NaO_3_ ([M + Na]^+^): 625.4340; found: 625.4339.

**Figure S17.** Key ^1^H ^1^H COSY and HMBC correlations of **9**.

**Isolation of (*R*)-ricinoleic acid from CO-FA S**

**Isolation.** The CO-FA S (1.0 g) was chromatographed on a silica gel column eluted with a gradient of hexane/ethyl acetate (9:1, 4:1, 2:1, 1:1, and 1:2) followed by a gradient of ethyl acetate/methanol (9:1 and 0:1) to give seven fractions; Fr. 1 (45.1 mg), Fr. 2 (462 mg), Fr. 3 (409 mg), Fr. 4 (17.5 mg), Fr. 5 (13.4 mg), Fr. 6 (62.4 mg), and Fr. 7 (22.4 mg). Then, a part of Fr. 3 was purified by preparative TLC eluted with hexane/ethyl acetate (sat. H_2_O) (7:3) to afford (*R*)-ricinoleic acid (9.98 mg) as a colorless oil.

${\boldsymbol{[}\boldsymbol{\alpha}\boldsymbol{]}}_{\mathbf{D}}^{\mathbf{26}}$ = +4.79° (*c* 0.30, acetone) [lit.^3^ [${\alpha]}_{D}^{26}$ = +7.15° (*c* 0.94, acetone, 97% purity)].

**^1^H NMR** (600 MHz, CDCl_3_): δ 0.87–0.90 (m, 3H), 1.26–1.51 (m, 18H), 1.61–1.66 (m, 2H), 2.05 (q, 2H, *J* = 7.2 Hz), 2.22 (t. 2H, *J* = 7.2 Hz), 2.34 (t, 2H, *J* = 7.2 Hz), 3.60–3.65 (m, 1H), 5.38–5.42 (m, 1H), 5.54–5.58 (m, 1H).

**^13^C NMR** (151 MHz, CDCl_3_): δ 14.0 (CH_3_), 22.6 (CH_2_), 24.7 (CH_2_), 25.6 (CH_2_), 27.3 (CH_2_), 29.0 (CH_2_), 29.0 (CH_2_), 29.1 (CH_2_), 29.3 (CH_2_), 29.5 (CH_2_), 31.8 (CH_2_), 34.0 (CH_2_), 35.0 (CH_2_), 36.5 (CH_2_), 125.2 (CH), 132.6 (CH), 178.7 (C).

**IR** (film): cm^-1^ 3421, 3118, 3010, 2928, 2856, 1712, 1459, 1404, 1282, 1079, 725.

**HRMS** (ESI-TOF): *m/z* calcd for C_18_H_34_NaO_3_ ([M + Na]^+^): 321.2400; found: 321.2403.

**Synthesis of 10–13**

**Extraction.** The dried rhizome powder of *Curcuma zedoaria* (CZ1, 50 g) was extracted with methanol (200 mL) at room temperature for 24 hours with vigorous stirring. The resulting solution was filtered and concentrated *in vacuo* to afford the extract (1.71 g).

**Ugi reaction.** To a solution of the methanol extract of *C. zedoaria* (1.71 g) in methanol (3.60 mL, 0.5 M), benzylamine (193 mg, 1.80 mmol) was added. The resulting mixture was stirred at room temperature for 1 hour. Then, castor oil fatty acids (CO-FA S) (590 μL) was added and the solution was cooled to 0 °C. Then, cyclohexyl isocyanide (197 mg, 1.80 mmol) was added and stirred for 1 hour. After vigorous stirring at room temperature for 7 days, the reaction mixture was concentrated *in vacuo* to give the crude mixture (2.58 g).

**Isolation.** The engineered extract was chromatographed on a silica gel column eluted with a gradient of hexane/ethyl acetate (9:1, 2:1, 1:1, and 1:2) followed by a gradient of ethyl acetate/methanol (9:1 and 0:1) to give six fractions; Fr. 1 (465 mg), Fr. 2 (954 mg), Fr. 3 (455 mg), Fr. 4 (307 mg), Fr. 5 (694 mg), and Fr. 6 (676 mg). Fr. 3 (451 mg) was re-chromatographed on a silica gel column eluted with a gradient of hexane/ethyl acetate (3:2 to 1:4) to give nine fractions; Fr. 3.1 (7.87 mg), Fr. 3.2 (22.2 mg), Fr. 3.3 (21.4 mg), Fr. 3.4 (20.2 mg), Fr. 3.5 (89.2 mg), Fr. 3.6 (37.3 mg), Fr. 3.7 (29.1 mg), Fr. 3.8 (36.3 mg), and Fr. 3.9 (76.4 mg). A combined mixture of Fr. 3.5 and Fr. 3.6 were purified by preparative TLC eluted with CHCl_3_/ethyl acetate (7:3) to give a 1:1 diastereomeric mixture of **10** (53.8 mg) as a colorless amorphous solid. Fr. 3.2 was then purified by preparative TLC eluted with CHCl_3_/methanol (99:1) to give **9** (12.3 mg) a colorless amorphous solid. A combined mixture of Fr. 3.1 and Fr. 3.2.1 was purified by preparative TLC eluted with CHCl_3_/ethyl acetate (9:1) to give an inseparable mixture of **12** and **13** (0.73 mg). Fr. 3.3 was then purified by preparative TLC eluted with CHCl_3_/methanol (49:1) to give four fractions; Fr. 3.3.1 (4.22 mg), Fr. 3.3.2 (0.88 mg), Fr. 3.3.3 (1.33 mg), and Fr. 3.3.4 (1.92 mg). Fr. 3.3.1 was repurified by preparative TLC eluted with dichloromethane/2-propanol (49:1) to give **11** (1.84 mg) as a colorless oil. Fr. 3.3.4 was also purified by preparative TLC eluted with dichloromethane/ethyl acetate (7:3) to give **11** (1.13 mg) as a colorless oil. Fr. 5 (690 mg) was chromatographed on a silica gel column eluted with CHCl_3_/methanol (99:1) to give four fractions; Fr. 5.1 (3.57 mg), Fr. 5.2 (20.2 mg), Fr. 5.3 (12.2 mg), and Fr. 5.4 (585 mg). Fr. 5.2 was repeatedly purified by preparative TLC eluted with CHCl_3_/diethyl ether (9:1) and then with dichloromethane/2-propanol (24:1) to give **5** (1.26 mg) a colorless amorphous solid.

**10**: (12*R*,*Z*)-*N*-Benzyl-*N*-(1-(cyclohexylamino)-2-methyl-4-((1*S*,6*R*,7*R*)-1-methyl-3-oxo-4-(propan-2-ylidene)bicyclo[4.1.0]heptan-7-yl)-1-oxobutan-2-yl)-12-hydroxyoctadec-9-enamide

**^1^H NMR** (600 MHz, CDCl_3_): δ 0.35–0.39 (m, 1H), 0.55–0.59 (m, 1H), 0.87 (t, 3H, *J* = 6.6 Hz), 0.98 and 1.02 (s, 3H, diastereomers), 1.06–1.48 (m, 25H), 1.34 and 1.35 (s, 3H, diastereomers), 1.54–1.72 (m, 6H), 1.76 and 1.77 (s, 3H, diastereomers), 1.88–1.92 (m, 2H), 1.99–2.05 (m, 3H), 2.07 (s, 3H), 2.17–2.31 (m, 4H), 2.42–2.53 (m, 2H), 2.71–2.81 (m, 2H), 3.57–3.61 (m, 1H), 3.70–3.77 (m, 1H), 4.50–4.62 (m, 2H), 5.36–5.40 (m, 1H), 5.50–5.56 (m, 2H), 7.25–7.28 (m, 1H), 7.35–7.38 (m, 2H), 7.44–7.47 (m, 2H).

**^13^C NMR** (151 MHz, CDCl_3_): δ 14.2 (CH_3_), 19.1 and 19.1 (CH_3_, diastereomers), 20.0 and 20.1 (C, diastereomers), 21.6 (CH_3_), 22.7 (CH_2_), 23.5 and 23.6 (CH_3_, diastereomers), 23.6 and 23.6 (CH_3_, diastereomers), 24.0 (CH_2_), 24.1 and 24.1 (CH, diastereomers), 24.5 and 24.6 (CH, diastereomers), 25.0 (CH_2_), 25.0 (CH_2_), 25.6 and 25.6 (CH_2_, diastereomers), 25.8 (CH_2_), 25.8 (CH_2_), 27.5 (CH_2_), 28.0 (CH_2_), 29.2 (CH_2_), 29.4 (CH_2_), 29.4 (CH_2_), 29.5 (CH_2_), 29.7 (CH_2_), 31.9 (CH_2_), 33.2 (CH_2_), 33.3 (CH_2_), 34.8 (CH_2_), 35.5 (CH_2_), 36.9 (CH_2_), 37.0 (CH_2_), 48.2 (CH_2_), 48.4 (CH), 49.0 (CH_2_), 65.1 and 65.2 (C, diastereomers), 71.6 (CH), 125.3 (CH), 126.3 (CH), 127.3 and 127.4 (CH, diastereomers), 128.2 and 128.2 (C, diastereomers), 129.0 and 129.0 (CH, diastereomers), 133.4 (CH), 139.3 (C), 147.5 and 147.6 (C, diastereomers), 173.4 and 173.4 (C, diastereomers), 174.5 (C), 201.8 and 201.8 (C, diastereomers).

**IR** (film): cm^-1^ 3423, 3361, 2928, 2854, 1656, 1512, 1452, 1405, 1369, 1077, 755, 730, 700.

**HRMS** (ESI-TOF): *m/z* calcd for C_47_H_74_N_2_NaO_4_ ([M + Na]^+^): 753.5541; found: 753.5543.

**Figure S18.** Key ^1^H ^1^H COSY and HMBC correlations of **10**.

**11**: (7*R*,*Z*)-18-(Benzyl(1-(cyclohexylamino)-2-methyl-4-((1*S*,6*R*,7*R*)-1-methyl-3-oxo-4-(propan-2-ylidene)bicyclo[4.1.0]heptan-7-yl)-1-oxobutan-2-yl)amino)-18-oxooctadec-9-en-7-yl (12*R*,*Z*)-12-hydroxyoctadec-9-enoate

**^1^H NMR** (600 MHz, CDCl_3_): δ 0.36–0.40 (m, 1H), 0.57–0.60 (m, 1H), 0.87 (t, 3H, *J* = 7.2 Hz), 0.88 (t, 3H, *J* = 7.2 Hz), 0.99 and 1.03 (s, 3H, diastereomers), 1.09–1.39 (m, 39H), 1.36 and 1.36 (s, 3H, diastereomers), 1.42–1.74 (m, 12H), 1.77 and 1.78 (s, 3H, diastereomers), 1.89–2.06 (m, 7H), 2.09 and 2.09 (s, 3H, diastereomers), 2.20–2.32 (m, 8H), 2.43–2.54 (m, 2H), 2.75–2.82 (m, 2H), 3.59–3.63 (m, 1H), 3.71–3.79 (m, 1H), 4.51–4.66 (m, 2H), 4.87 (quintet, 1H, *J* = 6.0 Hz), 5.28–5.33 (m, 1H), 5.38–5.46 (m, 2H), 5.53–5.57 (m, 2H), 7.28 and 7.28 (t, 1H, *J* = 7.2 Hz, diastereomers), 7.38 and 7.39 (t, 2H, *J* = 7.2 Hz, diastereomers), 7.46 and 7.47 (d, 2H, *J* = 7.2 Hz, diastereomers).

**^13^C NMR** (151 MHz, CDCl_3_): δ 14.2 (CH_3_), 14.2 (CH_3_), 19.1 and 19.1 (CH_3_, diastereomers), 20.0 and 20.2 (C, diastereomers), 21.6 (CH_3_), 22.7 (CH_2_), 22.8 (CH_2_), 23.6 and 23.6 (CH_3_, diastereomers), 23.6 and 23.7 (CH_3_, diastereomers), 24.0 (CH_2_), 24.1 and 24.1 (CH, diastereomers), 24.6 and 24.6 (CH, diastereomers), 25.1 (CH_2_), 25.1 (CH_2_), 25.2 (CH_2_), 25.5 (CH_2_), 25.6 and 25.7 (CH_2_, diastereomers), 25.8 (CH_2_), 25.9 (CH_2_), 27.5 (CH_2_), 27.5 (CH_2_), 28.1 (CH_2_), 29.3 (CH_2_), 29.3 (CH_2_), 29.3 (CH_2_), 29.3 (CH_2_), 29.3 (CH_2_), 29.5 (CH_2_), 29.5 (CH_2_), 29.7 (CH_2_), 29.8 (CH_2_), 29.9 (CH_2_), 31.9 (CH_2_), 32.0 (CH_2_), 32.1 (CH_2_), 33.3 (CH_2_), 33.3 (CH_2_), 33.8 (CH_2_), 34.8 (CH_2_), 34.9 (CH_2_), 35.5 (CH_2_), 36.9 (CH_2_), 37.0 (CH_2_), 48.2 (CH_2_), 48.4 (CH), 49.1 (CH_2_), 65.2 and 65.3 (C, diastereomers), 71.7 (CH), 73.8 (CH), 124.5 (CH), 125.4 (CH), 126.3 (CH), 127.4 and 127.4 (CH, diastereomers), 128.2 and 128.3 (C, diastereomers), 129.0 and 129.1 (CH, diastereomers), 132.7 (CH), 133.5 (CH), 139.3 (C), 147.6 and 147.7 (C, diastereomers), 173.5 (C), 173.7 (C), 174.6 (C), 201.9 and 201.9 (C, diastereomers).

**IR** (film): cm^-1^ 3423, 2926, 2854, 1731, 1656, 1511, 1452, 1405, 1375, 1192, 1077, 801, 753, 728, 700.

**HRMS** (ESI-TOF): *m/z* calcd for C_65_H_106_N_2_NaO_6_ ([M + Na]^+^): 1033.7943; found: 1033.7943.

**Figure S19.** Key ^1^H ^1^H COSY and HMBC correlations of **11**.

**4. References**

(1) Dong, C., Uematsu, A., Kumazawa, S., Yamamoto, Y., Kodama, S., Nomoto, A., Ueshima, M., Ogawa, A. 2,4,6-Trihydroxybenzoic acid-catalyzed oxidative Ugi reactions with molecular oxygen via homo- and cross-coupling of amines. *J. Org. Chem.* **84**, 11562–11571 (2019).

(2) Tomohara, K., Ito, T., Hasegawa, N., Kato, A., Adachi, I. Direct chemical derivatization of natural plant extract: straightforward synthesis of natural plant-like hydantoin. *Tetrahedron Lett.* **57**, 924–927 (2016).

(3) Brown, J. B., Green, N. D. Studies on the chemistry of the fatty acids. V. The preparation of methyl ricinoleate and ricinoleic acid by fractional crystallization procedure. *J. Am. Chem. Soc.* **62**, 738–740 (1940).

**Figure S20.** ^1^H NMR spectrum (600 MHz) of **1a** in CDCl_3_.

**Figure S21.** ^13^C NMR spectrum (151 MHz) of **1a** in CDCl_3_.

**Figure S22.** ^1^H NMR spectrum (600 MHz) of **1b** in CDCl_3_.

**Figure S23.** ^1^H NMR spectrum (600 MHz) of **1c** in CDCl_3_.

**Figure S24.** ^13^C NMR spectrum (151 MHz) of **1c** in CDCl_3_.

**Figure S25.** ^1^H NMR spectrum (600 MHz) of **1d** in CDCl_3_.

**Figure S26.** ^13^C NMR (151 MHz) of **1d** in CDCl_3_.

**Figure S27.** ^1^H NMR spectrum (600 MHz) of **1e** in CDCl_3_.

**Figure S28.** ^13^C NMR spectrum (151 MHz) of **1e** in CDCl_3_.

**Figure S29.** ^1^H NMR spectrum (600 MHz) of **1f** in CDCl_3_.

**Figure S30.** ^13^C NMR spectrum (151 MHz) of **1f** in CDCl_3_.

**Figure S31.** ^1^H NMR spectrum (600 MHz) of **1g** in CDCl_3_.

**Figure S32.** ^13^C NMR spectrum (151 MHz) of **1g** in CDCl_3_.

**Figure S33.** ^1^H NMR spectrum (600 MHz) of **1h** in CDCl_3_.

**Figure S34.** ^13^C NMR spectrum (101 MHz) of **1h** in CDCl_3_.

**Figure S35.** ^1^H NMR spectrum (600 MHz) of **1i** in CDCl_3_.

**Figure S36.** ^13^C NMR spectrum (151 MHz) of **1i** in CDCl_3_.

**Figure S37.** ^1^H NMR spectrum (600 MHz) of **1j** in CDCl_3_.

**Figure S38.** ^13^C NMR spectrum (151 MHz) of **1j** in CDCl_3_.

**Figure S39.** ^1^H NMR spectrum (600 MHz) of **2** in CDCl_3_

**Figure S40**. ^13^C NMR spectrum (151 MHz) of **2** in CDCl_3_.

**Figure S41.** ^1^H ^1^H COSY spectrum (400 MHz) of **2** in CDCl_3_.

**Figure S42.** HMQC spectrum of **2** in CDCl_3_.

**Figure S43.** HMBC spectrum of **2** in CDCl_3_.

**Figure S44.** ^1^H NMR spectrum (400 MHz) of **4** in CDCl_3_.

**Figure S45.** ^13^C NMR spectrum (151 MHz) of **4** in CDCl_3_.

**Figure S46.** ^1^H ^1^H COSY spectrum (600 MHz) of **4** in CDCl_3_.

**Figure S47.** HMQC spectrum of **4** in CDCl_3_.

**Figure S48.** HMBC spectrum of **4** in CDCl_3_.

**Figure S49.** ^1^H NMR spectrum (600 MHz) of **5** in CDCl_3_.

**Figure S50.** ^13^C NMR spectrum (151 MHz) of **5** in CDCl_3_.

**Figure S51.** ^1^H ^1^H COSY spectrum (600 MHz) of **5** in CDCl_3_.

**Figure S52.** HMQC spectrum of **5** in CDCl_3_.

**Figure S53.** ^1^H NMR spectra (600 MHz) of **7a** in CDCl_3_.

**Figure S54.** ^13^C NMR spectrum (151 MHz) of **7a** in CDCl_3_.

**Figure S55.** ^1^H ^1^H COSY spectrum (600 MHz) of **7a** in CDCl_3_.

**Figure S56**. HMQC spectrum of **7a** in CDCl_3_.

**Figure S57.** HMBC spectrum of **7a** in CDCl_3_.

**Figure S58.** ^1^H NMR spectrum (600 MHz) of **7b** in CDCl_3_.

**Figure S59.** ^13^C NMR spectrum (151 MHz) of **7b** in CDCl_3_.

**Figure S60.** ^1^H ^1^H COSY spectrum (600 MHz) of **7b** in CDCl_3_.

**Figure S61.** ^1^H NMR spectrum (600 MHz) of **7c** in CDCl_3_.

**Figure S62.** ^13^C NMR spectrum (151 MHz) of **7c** in CDCl_3_.

**Figure S63.** ^1^H ^1^H COSY spectrum (600 MHz) of **7c** in CDCl_3_.

**Figure S64.** ^1^H NMR spectrum (600MHz) of **8a** in CDCl_3_.

**Figure S65.** ^13^C NMR spectrum (151 MHz) of **8a** in CDCl_3_.

**Figure S66.** ^1^H ^1^H COSY spectrum (600 MHz) of **8a** in CDCl_3_.

**Figure S67.** HMQC spectrum of **8a** in CDCl_3_.

**Figure S68.** HMBC spectrum of **8a** in CDCl_3_.

**Figure S69.** ^1^H NMR spectrum (600 MHz) of **8b** in CDCl_3_.

**Figure S70.** ^13^C NMR spectrum (151 MHz) of **8b** in CDCl_3_.

**Figure S71.** ^1^H ^1^H COSY spectrum (600 MHz) of **8b** in CDCl_3_.

**Figure S72.** HMQC spectrum of **8b** in CDCl_3_.

**Figure S73.** HMBC spectrum of **8b** in CDCl_3_.

**Figure S74.** ^1^H NMR spectrum (600 MHz) of **8c** in CDCl_3_.

**Figure S75.** ^13^C NMR spectrum (151 MHz) of **8c** in CDCl_3_.

**Figure S76.** ^1^H ^1^H COSY spectrum (600 MHz) of **8c** in CDCl_3_.

**Figure S77.** HMQC spectrum of **8c** in CDCl_3_.

**Figure S78.** HMBC spectrum of **8c** in CDCl_3_.

**Figure S79.** ^1^H NMR spectrum (600 MHz) of **8d** in CDCl_3_.

**Figure S80.** ^13^C NMR spectrum (151 MHz) of **8d** in CDCl_3_.

**Figure S81.** ^1^H ^1^H COSY spectrum (600 MHz) of **8d** in CDCl_3_.

**Figure S82.** HMQC spectrum of **8d** in CDCl_3_.

**Figure S83.** HMBC spectrum of **8d** in CDCl_3_.

**Figure S84.** ^1^H NMR spectrum (600 MHz) of **9** in CDCl_3_.

**Figure S85.** ^13^C NMR spectrum (151 MHz) of **9** in CDCl_3_.

**Figure S86.** ^1^H ^1^H COSY spectrum (600 MHz) of **9** in CDCl_3_.

**Figure S87.** HMQC spectrum of **9** in CDCl_3_.

**Figure S88.** HMBC spectrum of **9** in CDCl_3_.

**Figure S89.** ^1^H NMR spectrum (600 MHz) of (*R*)-ricinoleic acid in CDCl_3_.

**Figure S90.** ^13^C NMR spectrum (151 MHz) of (*R*)-ricinoleic acid in CDCl_3_.

**Figure S91.** ^1^H NMR spectrum (600 MHz) of **10** in CDCl_3_.

**Figure S92.** ^13^C NMR spectrum (151 MHz) of **10** in CDCl_3_.

**Figure S93.** ^1^H ^1^H COSY spectrum (600 MHz) of **10** in CDCl_3_.

**Figure S94.** HMQC spectrum of **10** in CDCl_3_.

**Figure S95.** HMBC spectrum of **10** in CDCl_3_.

**Figure S96.** ^1^H NMR spectrum (600 MHz) of **11** in CDCl_3_.

**Figure S97.** ^13^C NMR spectrum (151 MHz) of **11** in CDCl_3_.

**Figure S98.** ^1^H ^1^H COSY spectrum (600 MHz) of **11** in CDCl_3_.

**Figure S99.** HMQC spectrum of **11** in CDCl_3_.

**Figure S100.** HMBC spectrum of **11** in CDCl_3_.
